# Growth of *Porphyromonas gingivalis* on human serum albumin triggers programmed cell death

**DOI:** 10.1080/20002297.2022.2161182

**Published:** 2022-12-22

**Authors:** Shirin Ghods, Mohammad F. Moradali, Danielle Duryea, Alejandro R. Walker, Mary E. Davey

**Affiliations:** Department of Oral Biology, College of Dentistry, University of Florida, Gainesville, FL, USA

**Keywords:** Lytic transglycosylase, polyamines, autolysis, *p. gingivalis*, asRNA

## Abstract

**Aims:**

Gingival crevicular fluid (GCF) constitutes the primary growth substrate for *Porphyromonas gingivalis in vivo*. The goal of this work was to evaluate the growth of different strains of *P. gingivalis* on human serum albumin (HSA), a major constituent of GCF.

**Methods:**

Growth of five different strains of *P.* *gingivalis* in the HSA medium was examined and, surprisingly, three of the strains underwent autolysis within 24 h. Comparative transcriptomic analysis was used to identify genes involved in autolysis.

**Results:**

Two highly related reference strains (W50 and W83) differed dramatically in their survival when grown on HSA. Strain W83 grew fast and lysed within 24 h, while W50 survived for an additional 20 h. Differential gene expression analysis led us to a gene cluster containing enzymes involved in arginine metabolism and a gene predicted to be lytic murein transglycosylase, which are known to play a role in autolysis. Deletion of this gene (PG0139) resulted in a mutant that did not lyse, and complementation restored the HSA lysis phenotype, indicating that this enzyme plays a central role in the autolysis of *P. gingivalis*.

**Conclusions:**

*P. gingivalis* undergoes autolysis when provided with HSA as a substrate for growth.

## Introduction

*Porphyromonas gingivalis* is a Gram-negative oral anaerobe implicated as an important etiological agent in periodontal diseases, a chronic inflammatory disease that can lead to destruction of the tissues supporting the teeth and development of other systemic and chronic diseases [1–3]. This endogenous pathogen persists within a complex microbial community below the gum line in proximity to gingival tissue [4,5]. A large set of *P. gingivalis* strains have been isolated from clinical samples that differ in their degree of virulence and pathogenicity, as well as their ability to invade tissues and cells [6,7]. To date, the genomes of over 80 strains have been sequenced, and type strains have been categorized into two groups via phylogenomic analysis; the first group consists of strains ATCC 33277, 381, and HG66, while the other consists of W83, W50, and A7436 [8]. It has become increasingly clear that, due to extensive allelic variations in key pathogenicity genes, including genes involved in the biosynthesis of fimbriae and capsular polysaccharides, these strains differ in their phenotype, i.e. biofilm formation, encapsulation, and virulence. Fimbriated strains (e.g. 381 and ATCC 33277) can form robust biofilms, while encapsulated strains (e.g. W83, W50, and A7436) have been found to be weak colonizers [6,9–11]. *P. gingivalis* is asaccharolytic and highly proteolytic and must obtain its carbon, nitrogen, and energy from protein. It produces a wide variety of proteases, including the cysteine proteases known as gingipains (RgpA, RgpB, and Kgp) that account for >85% of the total proteolytic activity of *P. gingivalis* [12]. Intriguingly, free amino acids and sugars do not support growth [13–15]. Here, we sought to investigate growth on human serum albumin (HSA) with respect to utilization as a biologically relevant substrate that is abundantly contained (~35 mg/mL) in gingival crevicular fluid (GCF) [16]. Since the flow of GCF into the subgingival crevice has increased during disease progression [17], we reasoned that GCF provides high levels of HSA as a growth substrate for amino acid fermenting subgingival microbiota. Previous growth studies have shown that both HSA and bovine serum albumin (BSA) support *P. gingivalis* growth when combined with α-ketoglutarate [18,19], and biofilm formation in our HSA-based medium was recently reported [20]; yet to our knowledge planktonic growth on HSA as the sole carbon and energy sources has never been assessed. Importantly, although the structure of BSA and HSA are highly similar, the amino acid sequence is distinct [21]. Given its biological relevance, we hypothesized that HSA may be a preferred substrate and our results show that indeed HSA is readily metabolized by *P. gingivalis*. The data also demonstrated that growth rates and survival during the stationary phase of different types of strains vary widely when grown on HSA. Importantly, strain W83 and strain W50, two highly similar strains [22], demonstrated dramatically different growth phenotypes in this medium. Strain W83 grew at a faster rate during exponential growth and lysed soon after entering the stationary phase, while W50 survived, showing the long-term survival that is typically observed when *P. gingivalis* is grown in complex media. Differential gene expression analysis led us to a gene cluster containing enzymes involved in arginine metabolism and polyamine synthesis, and a gene predicted to be a soluble lytic murein transglycosylase (up to 53.25% identity), which are known to play a role in PCD (programmed cell death) by catalyzing the non-hydrolytic cleavage of peptidoglycan structures [23]. Overall, the findings show that *P. gingivalis* undergoes autolysis and even highly related strains can differ greatly in their physiological state when they encounter the nutritional conditions of the subgingival environment (high levels of HSA).

## Materials and methods

### Bacterial strains and culture conditions

*P. gingivalis* strains W83, W50, 381, ATCC 33277, and A7436 were used in this study. They were grown on Trypticase Soy Agar plates supplemented with 5 µg mL ^−1^ hemin, 1 µg mL^−1^ menadione, and 5% defibrinated sheep blood (BAPHK) (Northeast Laboratory Services) at 37°C in an anaerobic chamber (Coy Lab Products, Grass Lake, MI) with an atmosphere containing 5% hydrogen, 10% carbon dioxide, and 85% nitrogen for 4 days. Planktonic cultures of *P. gingivalis* as starter culture were grown in Todd Hewitt Broth (Becton, Dickinson and Company, Franklin Lakes, NJ) supplemented with 5 µg mL^−1^ hemin and 1 µg mL^−1^ menadione (THBHK) anaerobically for 24 h at 37°C, then sub-cultured into pre – reduced appropriate media to make OD ~ 0.25–0.3 to study the growth rate. In this study, 1% HyClone™ bovine serum albumin (denatured) (BSA) (GE Healthcare Life Sciences), 0.5% and 1% human serum albumin (Sigma-Aldrich, cat #: A9511) were separately applied in the basal salt (phosphate buffer) PESthat contained 14 mM Na_2_HPO_4_, 10 mM KCl, 10 mM MgCl_2_, pH 7.3 and were used as working media. All media were supplemented with 5 µg mL−^1^ hemin and 1 µg mL^−1^ menadione; supplemented BSA- and HSA-based media with hemin and menadione were named BSAHK and HSAHK, respectively.

### Growth analysis

*P. gingivalis* strains were grown anaerobically in a Coy anaerobic chamber on BAPHK and inoculated anaerobically in THBHK for 24 h. Cultures grown overnight in THBHK were centrifuged and the pellet was suspended in pre-reduced working medium (1% BSAHK, 0.5% and 1% HSAHK) supplemented with 5 μg mL^−1^ hemin, and 1 μg mL^−1^ menadione. Bacterial growth was then monitored by measuring the optical density at 600 nm (OD_600_) and presented as the mean ± standard deviations (n = 3). To estimate the number of viable bacteria, planktonic growth cultures were subsequently prepared at dilutions for plating on BAPHK plates and inoculated anaerobically for 4–5 days. The number of colony-forming units (CFU) of growth culture at different time points was determined.

### Growth analysis with filtered supernatant from W83 lysis phase cultures

Liquid cultures of *P. gingivalis* W83 were prepared as described above. Cultures were harvested at early and late lysis phases, centrifuged at 4,000 × g for 10 min, and then filtered (0.2 μm PES). A pre-reduced cell-free supernatant was supplemented with 5 μg mL^−1^ hemin, and 1 μg mL^−1^ menadione was used to re-suspend active cells of W83 and W50. Active cells were prepared as described in the growth rate assay; when the culture reached the mid-log phase (OD_600_: 0.5), cell cultures were centrifuged inside the anaerobic chamber, and the pellets were re-suspended in the cell-free supernatant to reach OD_600_: 0.5, and the growth over time was monitored. Results were presented as the mean ± standard deviations (n = 3).

### Construction of a W83 PG0139 deletion mutant and complementation

A PG0139 deletion mutant, W83ΔPG0139, was created through allelic exchange mutagenesis. As shown in Supplemental Figure S6, the gene PG0139 was replaced by an in-frame promoterless erythromycin resistance cassette. Overlapping genetic fragments were created using PCR and plasmid pUC19 as a vector. The fragments were attached by NEBuilder HiFi Assembly (New England Biolabs, Inc.), and the resulting recombinant plasmid was transformed into *Escherichia coli* strain DH5α and used for sequencing confirmation by Sanger sequencing at Genewiz, Inc. from Azenta Life Sciences (South Plainfield, NJ). The sequence-confirmed plasmid was isolated and used to amplify a linear DNA fragment with all necessary components for transformation by electroporation into the competent wild-type W83. Transformants were selected on BAPHK agar containing 10 µg/mL of erythromycin (Erm), and the genome sequence of candidate strains was confirmed.

Complementation with plasmid was unsuccessful, presumably because of the lytic transglycosylase activity of PG0139 and toxicity to the cell at plasmid copy number. Instead, complementation via knock-in was used via allelic exchange mutagenesis for a linear DNA fragment adding PG0139 back to the original genetic position and including a nonpolar tetracycline resistance cassette as an antibiotic selection marker (see Supplemental Figure S6). A plasmid was created by inserting this fragment into pUC19 using the NEBuilder HiFi Assembly and used as a template for amplification of the linear fragment of interest. Competent W83ΔPG0139 were generated and transformed with the knock-in cassette by electroporation. W83ΔPG0139-C mutant candidates were selected and grown on BAPHK agar containing 1 µg/mL of tetracycline and the sequence was confirmed. Lastly, growth on HSAHK of the parent strain W83, W83ΔPG0139, and the complemented strain (W83ΔPG0139-C) were examined, as described above.

### RNA extraction, sequencing, and bioinformatic analyses

Samples for RNA extraction were obtained from HSAHK broth cultures of strain W83 and W50 during three different phases of strain W83 growth [i.e. late exponential (12.5 h, sample A), early stationary (15.5 h, sample B), and what is referred to here as the early lysis phases (20 h, sample C)]. RNA was extracted from W50 cultures at the same time-points; yet strain W50 remained in exponential growth, and it did not enter the stationary phase and did not lyse. At least four replicates for each time-point were prepared for each set of samples (n = 4). Cells were disrupted using the Direct-zol™ RNA MiniPrep Kit (Zymo Research). The RNA extraction and preparation were performed as described previously [24,25]. Importantly, to prevent an oxidative stress response, the cultures were processed inside the anaerobic chamber. RNA samples were delivered to the Gene Expression and Genotyping core of ICBR at the University of Florida. Sample quality determination and sequencing were performed by the gene expression and genotyping core in the ICBR [25]. Deep sequencing was performed on the Illumina® NovaSeq 6000 system instrument using the clustering and sequencing reagents provided by Illumina®. The bioinformatics section of this work was conducted entirely in the High-Performance Cluster (HiperGator2) at the University of Florida. The quality control of the raw sequencing data, conducted with FastQC (Babraham Institute) revealed that the data had a consistent average Phred score of 36, which according to our experience did not require quality trimming and was parsed directly to mapping with the Burrows-Wheeler aligner [26] (BWA-mem). Sequencing data from both W83 and W50 strains were mapped against the reference genome *P. gingivalis* W83 (NCBI: NC_002950.2). This was to ensure a common feature list for both *P. gingivalis* strains. Alignment files were then sorted with samtools [27]. Counts of transcriptomes mapped to reference genes were conducted with Htseq-counts [28], and the output was then parsed to the R statistical program (https://www.r-project.org/) and analyzed with EdgeR [29]. During the differential expression analysis, we eliminated all feature/genes with a p-value >0.05 and a fold change of less than 2.0.

A parallel differential expression analysis was conducted with Rockhopper [30]. In particular, we used this program to assess differential expression of antisense RNA (asRNA) transcripts. The Degust web tool (http://victorian-bioinformaticsconsortium.github.io/degust/) was also applied for differential expression of asRNA. Identification of differentially expressed genes and determining operonic organization as well as possible cognate metabolic and non-metabolic cellular processes were conducted using various bioinformatics databases, mainly including KEGG [31], BioCyc [32,33], and the National Center for Biotechnology Information (NCBI) databases (https://www.ncbi.nlm.nih.gov).

### qRT-PCR analysis

Samples for RNA extraction were obtained from HSAHK broth cultures of strain W83 and W50 during exponential growth (12.5 h), as described above, and qRT-PCR analysis was conducted as described previously [34,35]. Briefly, cDNA was generated from the same amount of RNA from each sample (1 µg) using the RNA to cDNA EcoDry PreMix Kit (Takara Bio, Inc.) according to the manufacturer’s protocol. cDNA was then diluted 10-fold and mixed with gene-specific primers and iQ SYBR Green Supermix (Bio-Rad). The qRT-PCR was performed using the CFX96 Real-Time System (Bio-Rad). Levels of the RNA transcript were normalized to the gene *recA*, as used previously in our laboratory [36].

## Results

### Utilization of human serum albumin (HSA) triggers P. gingivalis autolysis

**Figure 1.**
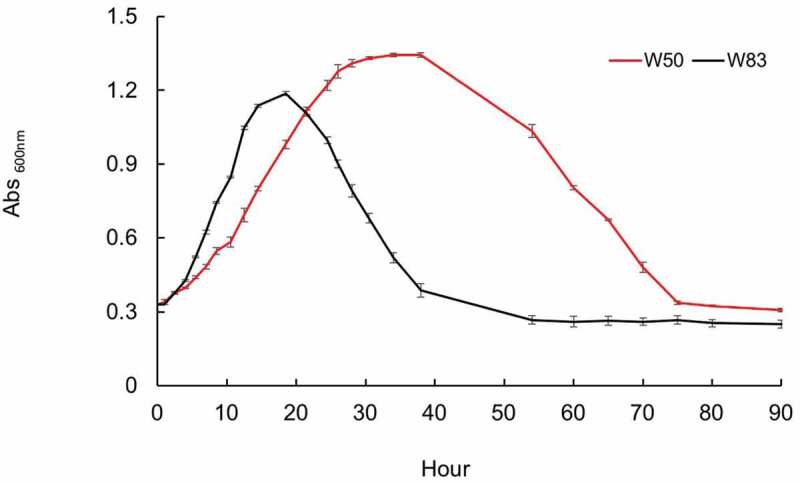
Growth rate of *P. gingivalis* strain W50 and W83 in 1% HSAHK medium. W83 grows exponentially with higher rate than the latter for up to 18 h to a maximum OD_600_ of ~1.2, then enters a short stationary phase (3–4 h) followed by a sharp cell lysis trend, leading to the complete cell lysis and medium clearance at about 35 h and designated as the fast lysis. W50 shows a lower exponential growth rate than W83 and undergoes a long steady stationary phase in which the cells withstand this condition for at least 40 h prior to a slower lysis trend. Data are representative of three replications (n = 3). Error bars represent the standard deviation of biological replicates.

Studies have shown that *P. gingivalis* grows well in the human serum or medium with BSA (or HSA) as a substrate in the presence of α-ketoglutarate as an electron (amino group) acceptor [19,20,25]. Here, we examined the growth of various strains of *P. gingivalis* on HSA, one of the most abundant proteinaceous substrates in GCF (Supplemental Figure S1) and discovered that cultures of strain W83 would clear (autolysis) soon after entering the stationary phase, yet its close relative strain W50 did not (Figure 1). Given this discovery, we hypothesized that various *P. gingivalis* strains may enter distinct physiological states at varying time points when grown on HSA, which activates programmed cell death. To test this hypothesis, we examined the growth curve phenotypes of various strains including W83 (encapsulated, non-fimbriated), W50 and A7436 (encapsulated, fimbriated), 381 (non-capsulated, highly fimbriated), and ATCC 33277 (non-capsulated, fimbriated) in response to growth in 1% HSA in a defined medium. Based on growth rate and colony forming unit (CFU) analyses, we observed that some strains (W83, 381, and ATCC 33277) demonstrated rapid-autolysis, growing at a faster rate during exponential growth for up to 18 h to a maximum OD_600_ of ~1.2, then entered a short stationary phase (3–4 h) followed by lysis and clearing of the culture after ~35 h of incubation (Table 1 and Supplemental Figure S1). The second group (strains W50 and A7436) displayed a slower exponential growth rate than the fast-lysis strains. These strains maintained a typical long steady stationary phase survival for at least 40 h followed by a slow lysis rate (Supplemental Figure S1). Quantification of CFUs for both strains did not detect live cells after lysis (Table 1).

**Table 1.** Colony-forming unit (CFU) of *P. gingivalis* strains W83 and W50 in 1% HSAHK medium.

	Time	OD_600_ average	CFU average
W83			
Start point	0	0.34	5.28E + 08
Late exponential	13.5	1.11	1.43E + 09
Early stationary	17.5	1.19	1.16E + 09
Early lysis	21	1.16	3.20E + 08
Late lysis	35.5	0.6	0.00E + 00
W50			
Start point	0	0.32	1.42E + 09
Mid exponential	17.5	0.92	2.40E + 09
Late stationary	37	1.26	1.67E + 09
mid lysis	51	1.05	6.63E + 06
Late lysis	72	0.48	0.00E + 00

Data are representative of three replications (n = 3). Error bars represent the standard deviation of technical replicates.

To test whether the heterogeneity in growth rate and lysis is a specific response to HSA availability, a similar assay was performed, yet BSA was provided as the growth substrate. As noted above, HSA and BSA have similar structures, yet display only 75.6% sequence homology and differ in binding affinity and specificity to other interacting molecules in biological systems [37,38]. To compare the growth on these two substrates, all selected strains were examined and found to have distinct growth curves when cultured in 1% BSA and compared to 1% HSA; from the fast-lysis group, W83 grew exponentially for about 50 h to a maximum OD_600_ of 1.81 followed by a very short stationary phase, that contrary to growth in the HSA-based medium, led to a gradual cell death. On the other hand, the other two members (381 and ATCC 33277) stopped logarithmic growth after 12 h of incubation, with the maximum OD_600_ of 0.91, which is contrary to their short stationary phase and fast lysis in the HSA-based medium (3–4 h). These two strains showed long-term survival in the stationary phase for up to 70 h in the BSA-based medium followed by gradual cell death (Supplemental Figure S2). Strains W50 and A7436 displayed a relatively similar exponential growth rate in the BSA-based medium but grew much slower than W83; W50 continued its logarithmic growth for up to 80 h after incubation followed by a short stationary phase and a gradual cell death, overall demonstrating a distinctly different growth curve in BSA versus the HSA-based medium (Supplemental Figure S2 and Supplemental Figure S1). In summary, these results show that although there is strain-to-strain variability, *P. gingivalis* has a specific response to the availability of HSA when compared to BSA or other commonly used complex medium. Importantly, the data suggest that unlike W83, strain W50 and strain A7436 appear to have a mechanism that limits their growth rate. Assessment of pH and cell morphology during growth on HSA did not reveal changes in these measurements for all strains tested.

To gain insight into the underlying mechanism of lysis, we hypothesized that the extracellular milieu of W83 cells at the lysis phase may contain lytic inducing by-products produced by HSA metabolism; hence, cell-free supernatant of the W83 lysis phase could potentially trigger lysis of the cells from the log phase of a fresh culture upon addition. To test this hypothesis, active cells of W83 and W50 strains (from the mid-exponential phase) were re-suspended in the cell-free supernatants collected from the early and late lysis steps of W83 cultures. This assay showed that both W83 and W50 retained the ability of exponential growth and viability for at least 12 h after the addition of the lysis supernatant (Supplemental Figure S3), indicating that neither extracellular lytic by-products nor nutrient depletion underlies the fast lysis of strain W83. In addition, the assessment of the growth rate of the capsule-null mutant W83 ΔPG0106 was similar to the parental strain, indicating that the heterogeneity in growth and lysis rate is not dependent on the *P. gingivalis* encapsulation (Supplemental Figure S4). Collectively, these assays indicate that external factors alone are not the driving force that triggers lysis and suggest that *P. gingivalis* has a regulated mechanism of programmed cell death.

### Transcriptomic study of P. gingivalis strain W83 compared to W50 when grown on HSA

**Figure 2.**
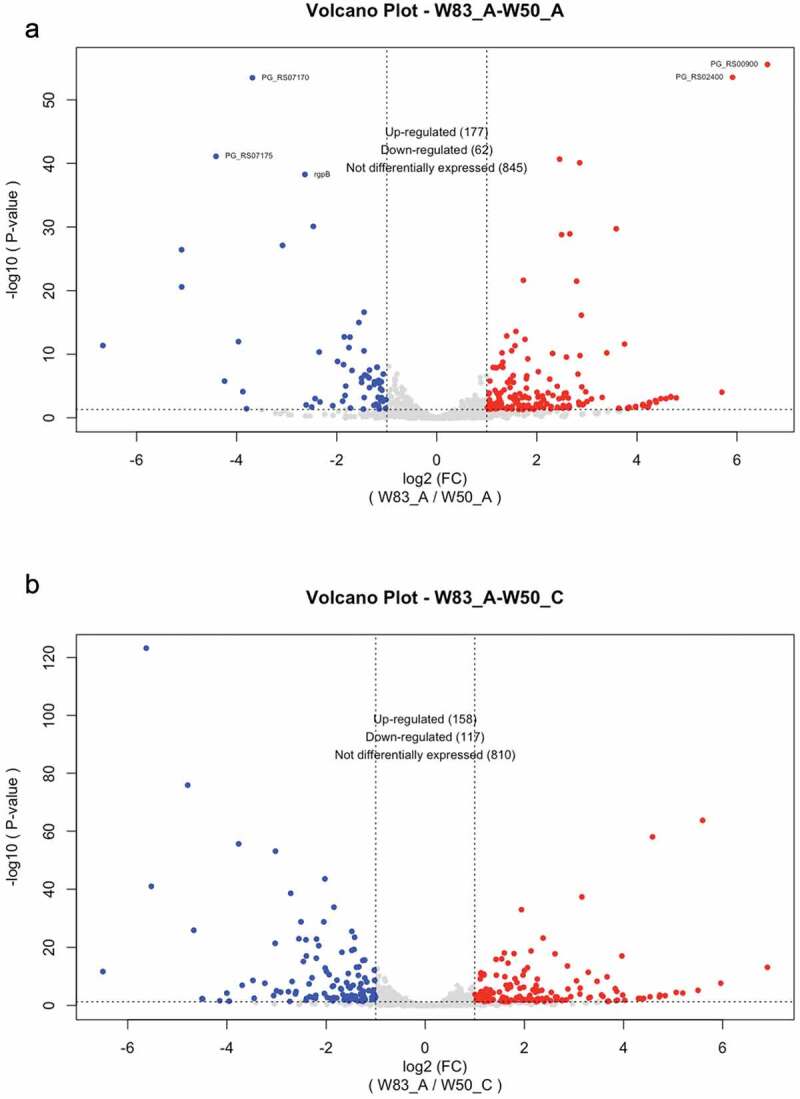
Differential gene expression in transcriptomic comparison of different growth phases of strain W83 compared to W50 when grown in 1% HSAHK. The volcano plots represent differential gene expression on a field of log_2_ fold change (FC) versus the negative log_10_ of the p-values of each gene. (a) Differential gene expression between the two strains at 12.5 h (A), exponential phase of growth. Briefly, there are 177 up-regulated genes and 62 down-regulated genes in strain W83 when compared to W50 during exponential growth. (b) Differential gene expression between strain W83 during exponential growth (12.5 h, A) and strain W50 during exponential growth (20 h, C) shows 158 activated genes and 117 down-regulations.

**Figure 3.**
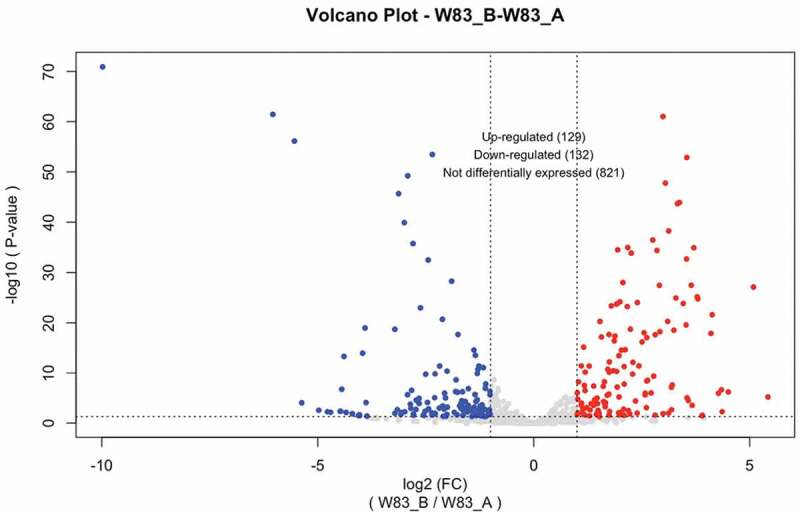
Differential gene expression in transcriptomic comparison of different growth phases of strain W83 when grown to stationary phase (15.5 h, B) compared to W83 cells in exponential phase of growth (12.5 h, A). The volcano-plots represent differential gene expression on a field of log_2_ fold change (FC) versus negative log_10_ of the p-values of each gene. Briefly, there are 129 genes expressed at higher levels and 132 genes expressed at lower levels in stationary phase cells when compared to cells in exponential phase.

In order to elucidate the mechanism-underlying autolysis and provide a better understanding of HSA metabolism, RNA-sequencing analysis was performed on RNA samples extracted from W83 and W50 during growth on HSA. Importantly, the genomes of these two strains are almost identical, hence we reasoned a comparison would reveal the mechanism(s) underlying autolysis. Based on the growth phases of the fast-lysis strain W83, pairwise RNA-Sequencing analyses during exponential growth were conducted. Additional growth phases of W83 cultures, i.e. early stationary (15.5 h) were also compared with the late exponential phase (12.5 h) (Figure 1). Considering almost identical genomes of W83 and W50 [39], a pairwise transcriptomic comparison (EdgeR analysis) of exponentially growing W83 and W50 cells at the same time-point (12.5 h), was compared (see Figure 2(a)) and showed that 21.9% (upregulated: 177; downregulated: 62), of the total annotated genes were differentially expressed at 2-fold or more. When the transcriptome of W83 at 12.5 h was compared with the transcriptome of W50 at 20 h (both in late exponential growth) the analysis revealed many of the same genes as the 12.5 h (W83A-W50A) comparison with 25.3% (upregulated; 158; downregulated: 62) of the total annotated genes being differentially expressed. In addition, as shown in Figure 3, pairwise transcriptomic analysis of early stationary (W83 C) versus late exponentially (W83 A) grown cells showed that 24.0% (upregulated: 129; downregulated: 132), of the total annotated genes were differentially expressed at 2-fold or more. Interestingly, some of the most differentially expressed transcripts were antisense RNAs (asRNAs); one hundred to many thousands of nucleotides in length that are transcribed from the opposing strand within specific annotated or unannotated genes. In particular, asRNAs were found to be differently expressed between the late exponential and lysis of W83, which showed 27 asRNAs expressed at lower levels (or absent) during the early lysis phase (Supplemental Table S2). Pairwise transcriptomic comparison of different growth phases of W83 cells along with a comparison of W83 with W50 during exponential phase are presented in Supplemental Table S2. By matching the given starts and ends of asRNA transcripts to the corresponding genomic regions and genes, we determined that in some cases two or more asRNAs corresponded to the sequence of one gene, while in other cases only one asRNA corresponded to a genomic region containing two or more consecutive genes (Supplemental Table S2). Overall, these findings indicated that anti-sense transcription of genes (which can be either negatively or positively influenced transcript levels) may play an important post-transcriptional regulatory role during growth-phase transition in HSA medium and appears to influence the levels of some of the most differentially expressed genes. Key findings from RNA-Sequence analyses are highlighted below.

### Differential expression of genes involved in envelope homeostasis, ppGpp signaling and arginine metabolism, in strain W83 are linked to autolysis

To begin our analysis, the transcriptomes of strain W83 were compared to W50 when they were both in exponential growth, i.e., W83 and W50 at 12.5 h, and W83 at 12.5 h compared to W50 at 20 h. As shown in Supplemental Tables S1a and S1b, EdgeR analysis showed that PG1648 and PG1808, which are two bifunctional ppGpp synthetases/hydrolases [40] were expressed at higher levels (2.9 FC and 4.9 FC, respectively) in strain W83 when compared to W50 in the exponential phase of growth, indicating that ppGpp signaling may play a key role in regulating autolysis. Higher expression levels were also detected for PG0195 (6.61 logFC), a rubrerythrin-family protein that is involved in the oxidative stress response [41,42], and PG1797 (4.68 logFC), a hybrid sensor/response regulator referred to as GppX, which is linked to regulation of gingipain expression and localization [43,44], as well as PG1421 (3.6 logFC), a predicted ferredoxin. Lastly, PG1780, encoding a serine palmitoyltransferase which is required for sphingolipid biosynthesis [45], was upregulated in strain W83 (2.3 logFC) along with PG0276 (2.3 logFC), which was recently shown to encode a ceramide synthase [46]. While the role of sphingolipids in *P. gingivalis* physiology is still under investigation, synthesis of these lipids was shown to play a role in oxidative stress resistance and stationary phase survival [45]. One particular area of the chromosome that was expressed at higher levels in strain W83 compared to W50 during exponential growth included the region from PG0136 to PG0153. Interestingly, this area contains a number of genes encoding enzymes involved in arginine metabolism and polyamine synthesis, including PG0144 (agmatine deiminase), PG0143 (amidohydrolase), and PG0152 (carboxyspermidine decarboxylase). This finding aligns with the higher expression levels (2.0 logFC in W83 at 12.5 h when compared to W50 at 20 h) of PG1424 the porphyromonas peptidylarginine deiminase (PPAD) that citrullinates arginine residues within peptides. In addition, at least two other genes in this area encode proteins known to be involved in peptidoglycan synthesis and remodeling, including PG0136 that encodes a putative flippase-like domain containing protein, and PG0139 encoding a putative lytic transglycosylase. Importantly, all of these differentially expressed genes were identified in both pairwise analyses, except PG1797 which was only found to be detected at higher levels in the 12.5 h comparison. Lastly, as shown in Supplemental Tables S3a and S3b, differentially expressed genes can be placed into various pathway classes involved in a variety of functions, including lipid metabolism, glycan synthesis, nucleotide metabolism, proper folding of proteins, translation, and amino acid metabolism.

**Figure 4.**
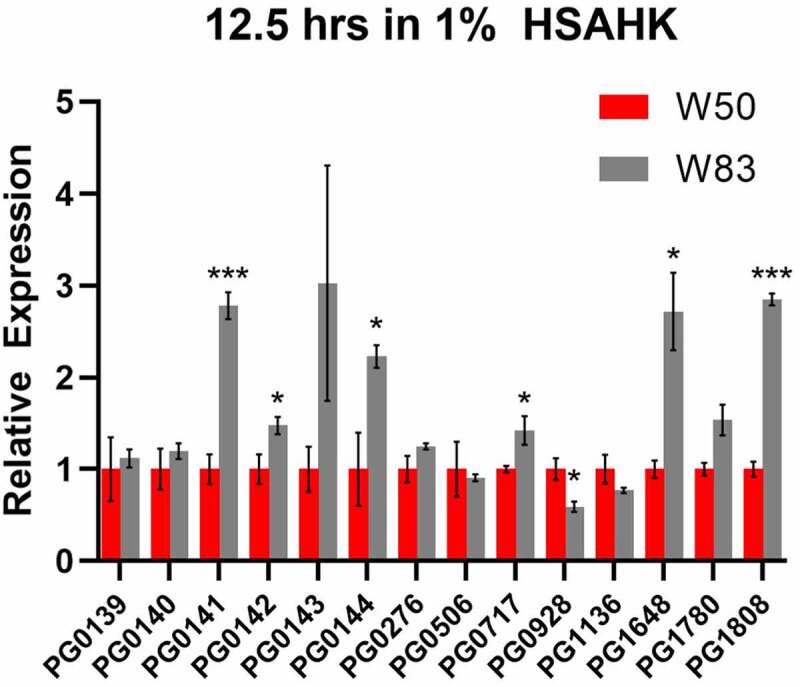
Quantitative PCR analysis of select genes after 12.5 h of growth in HSAHK. The genes selected were found to be differentially expressed by RNA-Sequencing at the same time point. The results are presented as the relative levels (mean ± s.D. of triplicate determinations) compared with the transcript levels of the strain W50.

Several genes identified by RNA-sequencing as differentially expressed were further examined by quantitative reverse transcription PCR (qRT-PCR) analysis by comparing RNA extracted from strain W83 and W50 at 12.5 h when grown on HSAHK. As shown in Figure 4, PG0144 (agmatine deiminase), was consistently expressed at higher levels in W83 when compared to W50, as well as PG0142, which is predicted to be co-transcribed with PG0139. Surprisingly, RT-qPCR did not detect differential expression of PG0139. The reason for the discrepancy between RNA-sequencing and RT-qPCR analysis is yet unclear and will require further study. Results also indicated a high degree of variability in the expression of PG0143 (amidohydrolase). While this variability was consistent throughout repeat experiments, the expression was consistently higher in strain W83 than W50. Lastly, the analysis confirmed higher levels of expression of the ppGpp synthase/hydrolase-encoding genes (PG1648 and PG1808) in strain W83 as compared to W50.

### Rockhopper analysis of strain W83 compared with strain W50 during exponential growth showed differential expression of antisense RNAs

As shown in Supplemental Table S2, asRNAs corresponding to the two iron-related gene products involved in oxidative stress discussed above [ferredoxin (PG1421) and rubrerythrin (PG0195)], along with PG1615 and PG1617 (putatively involved in succinate metabolism) and PG0537 (aminoacyl-histidine dipeptidase) were found to be highly expressed (up to 10.5-fold; *q*-value <0.01) during the exponential phase of W83 when compared with the same growth time-point of W50 cells and the corresponding genes (PG1421, PG0195, PG1615, PG1617, and PG0537) were also detected at higher levels, suggesting that the asRNA may support stability of these transcripts. In contrast, the initiation of W83 cell lysis in comparison to its late exponential phase was concomitant with more than twofold lower expression levels of a suite of asRNAs (27 transcripts) (Supplemental Table S2). Importantly, asRNAs detected at the lowest levels (by up to 10 times, *q*-value <0.01) in the early lysis phase of W83 cells correspond to the genes encoding HmuY (PG1551; heme-binding and iron acquisition), flavodoxin (PG1858; electron transport system), a predicted subunit K of v-type ATPase (PG1807; bioenergetics), aminoacyl-histidine dipeptidase/carnosinase PepD (PG0137), FabD (PG0138; fatty acid and phospholipid biosynthesis), and the arginine-specific gingipain RgpB (PG0506; nutrient acquisition).

Importantly, many of the corresponding genes on the sense strand were found to be differentially expressed with PG1551, PG1858, PG1807, and PG0506 being expressed at lower levels in W83 cells at 12.5 h (exponential growth) when compared with W83 at 20 h (cessation of growth) and when W83 was compared to W50 during exponential growth, suggesting that these asRNAs may play a role in stabilizing these transcripts; moreover, that higher expression levels of these antisense RNAs in strain W50 correlate with continued exponential growth of strain W50 when it is growing on HSA.

### Nutrient uptake rate and hemin availability direct the mode of growth and lysis

**Figure 5.**
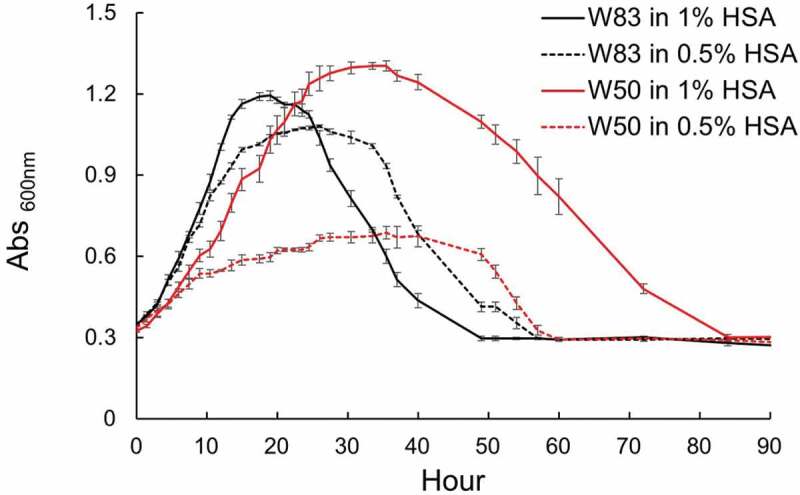
Analysis of the growth rate of W83 and W50 in the presence of different concentrations of HSA (1% and 0.5%). W83 shows higher rate of logarithmic growth in 1% HSAHK and reaches a higher turbidity than in 0.5% HSAHK. While W50 displays a much slower logarithmic growth compared to W83 in both concentrations of HSA and reaches a higher turbidity in 1% HSAHK. W83 grown in 0.5% HSAHK persists in stationary phase for a longer period of time when compared to growth in 1% HSAHK. It also shows a slower rate of lysis, indicating that logarithmic growth rate has negative correlation with the length of the stationary phase, and a positive correlation with the rate of cell lysis. Data are representative of three replications (n = 3). Error bars represent the standard deviation of biological replicates.

Since our findings showed that W83 displayed higher rates of logarithmic growth (Figure 1) and expression of higher levels of transporters and peptidases/proteases compared with W50 (Figure 2 and Supplemental Tables S1a and S1b), we postulated that the fast-lysis phenotype was linked to higher proteolytic activity and a higher rate of nutrient uptake. To begin to test this notion, we reversed our experimental design by using different concentrations of HSA (1% and 0.5%) in assessment of the growth rate. W83 showed higher rate of logarithmic growth in 1% HSA and reached a slightly higher turbidity than in 0.5% HSA (OD_600(1%HSA)_: 1.19 versus OD_600(0.5%HSA)_: 1.08). However, W83 growing in 1% HSA entered the stationary phase at 18 h that lasted in this stage for 1.5 h prior to a sharp lysis, while when growing in 0.5% HSA, W83 entered the stationary phase after 24 h and sustained viability for almost 3 h prior to a slower lysis rate (Figure 5), indicating that logarithmic growth rate has a negative correlation with the length of the stationary phase and positive correlation with the rate of cell lysis. On the other hand, W50 displayed a much slower growth rate during the exponential phase compared to W83 in both concentrations of HSA, but reached a higher turbidity in 1% HSA (OD_600(1%HSA)_: 1.3) after 33 h. However, in 0.5% HSA, W50 grew more slowly (15 h; OD_600(0.5%HSA)_: 0.67) than W83 and persisted at the same density for almost 15 h until cell death occurred.

Studies have shown that the peptidylarginine deiminase PPAD (PG1424) and RagA (PG0185) oligopeptide transporter, respectively, play important roles in maintaining an optimal extracellular proteolysis rate and oligopeptide uptake [47,48]. Since our current findings indicated that the rate of nutrient uptake and proteolytic activity positively correlated with autolysis and PPAD was found to be expressed at higher levels in strain W83 during exponential growth, we used the mutants 381△*ppad* and 381△*ragA* to investigate whether their defective functional characteristics may impact growth rate and cell death. Assessment of the growth rates showed that both mutants displayed a slower growth rate during the exponential phase, as expected, and a significant delay in transition to the stationary phase, along with a longer period of survival in the stationary phase when compared with the parental strain (Supplemental Figure S5a and b). This finding supported our notion that the physiological state of various strains of *P. gingivalis* is determined by the availability of proteinaceous substrates and the rate of the proteolytic activity and nutrient uptake.

**Figure 6.**
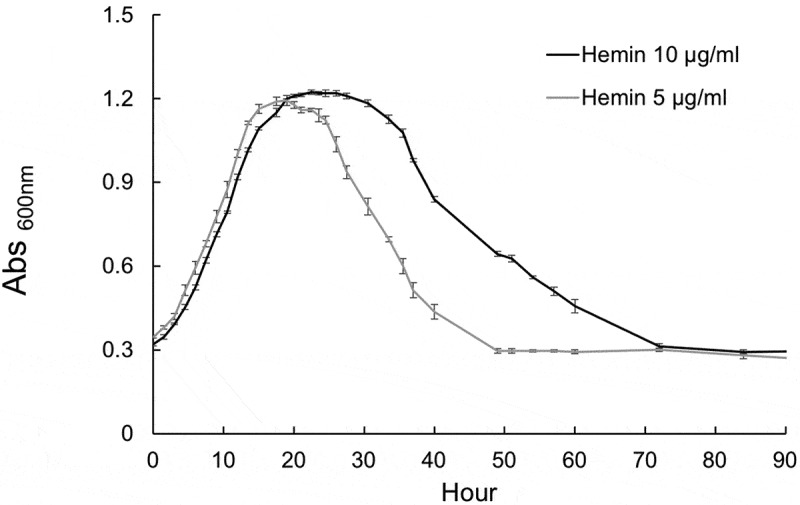
Analysis of the growth rate of W83 in the presence of different concentrations of hemin (10 and 5 μg/ml) as iron source when grown in 1% HSAHK. The exponential growth rate of W83 did not change in response to different concentrations of hemin, but higher concentration of hemin expanded the time window of the stationary phase and delayed the start time of cell lysis when compared with the lower concentration of hemin. Data are representative of three replications (n = 3). Error bars represent the standard deviation of biological replicates.

Furthermore, in addition to their role in generating peptide substrates for uptake, the gingipains through their degradation of hemoglobin and heme binding play a central role in the acquisition of iron from hemoglobin. Iron, in the form of heme, is an essential nutrient for survival of *P. gingivalis*. Once released from hemoglobin, both outer membrane receptors with high affinity for heme and heme binding by the gingipains promote sequestration and uptake [49,50]. Since our transcriptomic data indicated that the initiation of autolysis correlates with transcriptomic changes in iron acquisition pathways, we hypothesized that heme availability may be an important signal that influences the time window of the stationary phase and long-term survival. To test this hypothesis, we applied 5 µg/mL (typical concentration for *P. gingivalis* growth) and 10 µg/mL of hemin as an iron source in the presence of 1% HSA and the growth rates of W83 cultures were monitored for 90 h. Interestingly, the exponential growth rate of W83 did not change in response to different concentrations of hemin, but higher concentration of hemin expanded the time window of the stationary phase and delayed the lysis of cells for almost 10 h when compared with the lower concentration of hemin (Figure 6), indicating heme accessibility is a key factor that delays autolysis.

### The W83ΔPG0139 mutant does not undergo autolysis

**Figure 7.**
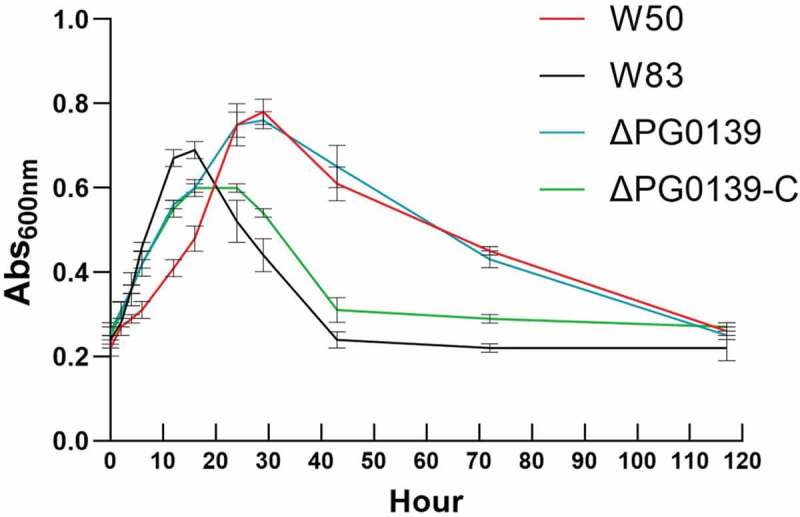
The autolysis defect in strain W83△ PG0139 was restored by complementation. W83 and its derivatives were grown in HSAHK for five days and the absorbance was monitored. W83 showed higher rate of logarithmic growth when compared to W50 and W83△PG0139 and the complemented strain W83△PG0139-C demonstrated a lysis phenotype, similar to the parent strain W83. Data are representative of three replications (n = 3). Error bars represent the standard deviation of biological replicates.

Lytic transglycosylases are involved in peptidoglycan recycling and remodeling by cleaving at *N*-acetylglucosamine (GlcNAc) and *N*-acetylmuramic acid (MurNAc) glycosidic bonds, releasing 1,6-anhydro-*N*-acetlMurNAc muropeptide products. This activity is known to contribute to mechanisms of PCD [51]. Because of this, PG0139, encoding a putative lytic-transglycosylase D, was chosen as a target to investigate the PCD that *P. gingivalis* demonstrates while using HSA as a substrate. A PG0139 knock-out, W83ΔPG0139, expressing erythromycin resistance was created. As shown in Figure 7, analysis of the growth of this strain on HSAHK revealed a growth pattern that excluded the rapid autolysis phase. The initial exponential growth rate, although slightly slower than that of wild-type W83, was still faster than that of W50. At the time-point when the wild-type strain W83 would normally lyse (~20 h), the knock-out strain continued to grow for up to 30 h of the total growth before slowly lysing. Complementation was attempted by plasmids, but unsuccessfully, presumably because of the toxic effects of the lytic transglycosylase at plasmid copy number. Instead, a knock-in strain was created that replaced PG0139 back into its original genetic position and added a tetracycline resistance cassette as an antibiotic selection marker. This strain, W83ΔPG0139-C, grew very similarly to the mutant strain until a time-point at approximately 18 h, where it enters a brief stationary phase. It then displayed rapid autolysis at approximately 22–24 h, just after the wild-type strain. The complementation revealed that the changes in autolysis in the knock-in strain are, in fact, due to PG0139 deletion. These results indicate that the lytic transglycosylase encoded by PG0139 plays an important role in autolysis.

## Discussion

Bacterial autolysis, self-digestion of the cell wall, represents a mechanism of programmed cell death [51]. This process is known to be the result of an internal mechanism that can be activated by internal and/or external cues. This study revealed that the Gram-negative oral anaerobe *P. gingivalis* undergoes PCD when grown on human serum albumin (HSA), and that growth and survival of various strains of *P. gingivalis* differ greatly when HSA is provided as a growth substrate. Transcriptomic analysis revealed that a predicted BamD-encoding gene (PG1215) is one of the most upregulated genes. BAM complex components are highly conserved across Gram-negative bacteria and essential to envelope biogenesis and maintenance by mediating proper folding and localization of outer membrane proteins; hence, the BAM complex is tightly regulated and essential to bacterial viability during all stages of growth and pathogenesis [52]. The analysis also revealed that an operon (PG0139-PG0142) containing a number of genes that encode enzymes involved in arginine metabolism and polyamine synthesis, including amidohydrolase (PG0143), agmatine deiminase (PG0144), and carboxyspermidine decarboxylase (PG0152) were expressed at higher levels in strain W83 during exponential growth when compared to W50. Increasing evidence has revealed that polyamines increase acid resistance in *E. coli* (76) and regulate biofilm formation in multiple species, including *Yersinia pestis* (77), *Bacillus subtilis* (78), and *E. coli*. In *Streptococcus pneumoniae*, it was also found that deletion of a gene for polyamine synthesis, an arginine decarboxylase, reduced the amount of capsular polysaccharide produced by some strains (82) and most relevant to this study, delayed autolysis [53]. The differences seen in these polyamine mutants have been mostly attributed to defects in gene expression regulation by polyamines. Therefore, it is possible that lower levels of RgpB expression in strain W83 results in a decrease in available arginine, which results in changes in the levels of polyamines, which ultimately controls the process of autolysis, but this needs further analysis. Importantly, this locus also contains *parA*, a chromosome partitioning protein (PG0142) and a predicted soluble lytic murein transglycosylase (PG0139). Since these enzymes are known to play a role in autolysis, we generated a PG0139 deletion mutant that confirmed that this gene is required for autolysis. Since addition of the spent supernatant from strain W83 during lysis supported growth and did not induce lysis in strain W50 or W83 within 20 h, the data indicated that neither extracellular lytic by-products nor nutrient depletion underlies the fast lysis phenotype. Accordingly, we posit that the autolysis observed represents programmed cell death that is activated by an inside signal. In response to this signal, the expression of genes involved in maintaining envelope homeostasis, along with proper folding of critical membrane proteins required for iron and nutrition acquisition, biosynthesis of the lipid bilayer, and electron transport are affected. Further studies will help to elucidate the environmental parameters that regulate the expression of this lytic transglycosylase which appears to be central to HSA-elicited autolysis.

Although elucidating the regulatory pathway that controls autolysis is still under investigation, it is important to note that the transcriptomic analysis showed differential expression (low expression in strain W83) of *cis*-encoded regulatory asRNA molecules. For many years, it has been well-established that asRNAs are encoded on the DNA strand opposite coding regions and can form extensive base-pairing interactions with the corresponding sense RNA molecules. Hence, a common working model for many bacteria is that *cis*-encoded asRNAs are an important genome-wide posttranscriptional mechanism involved in inhibiting initiation of translation or control of mRNA degradation and/or mRNA transcription termination; however, their mechanistic roles are still not fully understood [54–57]. Unlike small regulatory RNAs, asRNAs may range in size from a few hundred to thousands of nucleotides in length, and their abundance can vary extensively [58]. Our work demonstrated that an array of asRNAs are synthesized by *P. gingivalis*, similar to what has been shown in *Pseudomonas aeruginosa* and *Staphylococcus aureus* [57,59]. As addressed by others, more than 80% of the genes of the *P. aeruginosa* genome have been found to have a propensity for antisense transcription, while the majority of sequencing reads map on the sense strand; therefore, sense or antisense transcription was found as a regulatory mechanism that are inversely mediated and modulated by transcription factors in response to different conditions [55,59]. Even though only a small number of growth conditions and mechanisms driving differential expression of antisense transcripts in bacteria have been investigated, our data indicated that at least 1.4% (~27 asRNAs) of the protein-coding genes of *P. gingivalis* strain W83 can undergo antisense regulation during different phases of growth. Previously, it was shown that about 6% of antisense transcripts of *S. aureus* and *B. subtilis* can be differentially expressed when growth and environmental conditions were altered [54,60].

Importantly, previous studies investigated different factors with the potential to trigger *P. gingivalis* autolysis. One study showed that deletion of a predicted cell wall hydrolase/autolysin in strain W83 (PG1048) disrupted cell division and promoted biogenesis of outer membrane vesicle when *P. gingivalis* was grown in a complex medium; yet autolysis was not observed [61]. Another study observed autolysis of *P. gingivalis* strain 33277 after three days of incubation in complex medium and lysis resulted in the release of high levels of RgpA (Arg-gingipain) activity into the culture supernatants [62]; yet the underlying mechanism of autolysis was not determined. Although our analysis did not detect differential expression of *rgpA*, it did show that *rgpB* is expressed at lower levels in strain W83 when compared to strain W50, thus regulation of RgpB activity (cleavage at arginine residue) and its impact on the levels of arginine (see below) may play a role in autolysis when *P. gingivalis* is grown on HSA.

In addition, asRNA corresponding to HmuY showed the lowest relative expression at early lysis phase in strain W83, suggesting that these growth conditions are inducing changes in the levels of HmuY transcript (PG1551) or protein. Although changes in the levels of PG1551 (HmuY) were not detected, other genes in the PG1551 operon (PG1553, PG1555, and PG1556) were found – to be expressed at higher levels (2.8–3.8-fold) in strain W83 when compared to strain W50 during exponential growth. HmuY is an important outer membrane associated heme-binding lipoprotein that plays an essential role in the acquisition of iron, a critical co-factor for electron transport, growth, and virulence [63,64]; hence, our data provided some insight into the role of iron in tolerating envelope stress and suggested iron-acquisition genes as potential targets for drug development. Iron is a key signal for oxidative stress responses and expression of outer membrane proteins in bacteria [65] and hemin are known to protect *P. gingivalis* from oxidative stress [66]. Previously, it was shown that iron uptake in the cyanobacterium *Anabaena* sp. PCC 7120 is regulated by a 2,200-nucleotide *cis*-asRNA via decreasing *furA* expression and translation [67]. Also, outer membrane receptor for ferrienterochelin and colicins can counteract the production of reactive oxygen species during the general stress response [68,69]. Here, we showed that upon providing a higher concentration of hemin, *P. gingivalis* can extend long-term survival in stationary phase when grown on HSA.

Lastly, it is also important to note that *P. gingivalis* persists within a complex biofilm community. From this perspective, its ability to undergo autolysis may be highly significant. Autolysis not only reduces cell numbers but at the same time, it provides nutrients to the rest of the community. Autolysis during biofilm development generates extracellular DNA, which plays an important role in the structural integrity of the extracellular matrix. Furthermore, autolysis, like phage-induced lysis drives phenotypic diversity, by forcing adaptation to dramatic environmental perturbations. Hence, although the regulation and underlying mechanism of autolysis remains to be determined, we propose that the findings presented here provide a foundation for considering a role of *P. gingivalis* autolysis in perturbing the subgingival biofilm community.

## Supplementary Material

Supplemental Material

## Data Availability

The authors declare that the data supporting the findings of this study are available within the paper and its supplementary information files. Raw sequencing data are available on the NCBI Sequence Read Archive (SRA) under accession number PRJNA911789. https://www.ncbi.nlm.nih.gov/bioproject/PRJNA911789
