# Supplementary material for: Growth of Porphyromonas gingivalis on human serum albumin triggers programmed cell death
Source: J Oral Microbiol. 2022 Dec 22;15(1):2161182. doi: 10.1080/20002297.2022.2161182 (PMC9788703; doi:10.1080/20002297.2022.2161182)
Supplement: Supplemental Material [file ZJOM_A_2161182_SM7719.zip › supplementary files/HSA_Figures Supplemental S1.pdf]

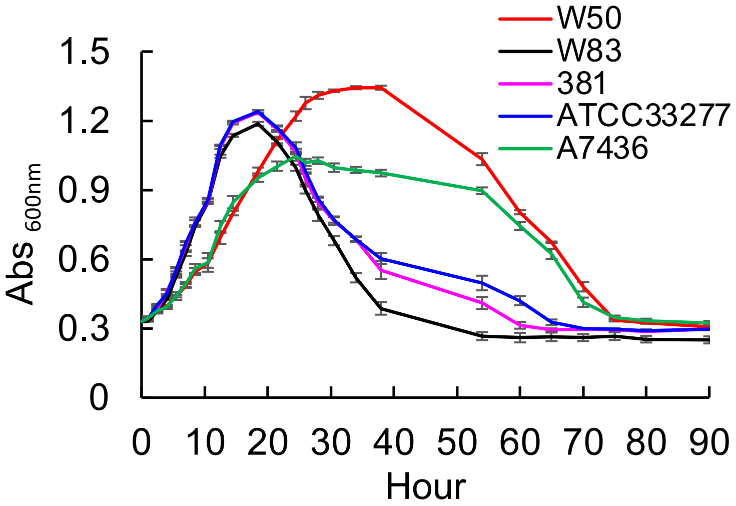

Figure S1. Growth rate of *P. gingivalis* strain W50, W83, 381, ATCC33277, and A7436 in 1% HSAHK medium. These strains are classified into two distinct groups designated as either fast- and slow lysis. The former including W83, 381, and ATCC 33277 grow exponentially with higher rate than the latter for up to 18 h to a maximum OD_{600} of ~1.2, then enter a short stationary phase (3-4 h) followed by a sharp cell lysis trend, leading to the complete cell lysis and medium clearance at about 35 h. The latter includes the strains W50 and A7436 showing a lower exponential growth rate than the fast-lysis group and undergo a long steady stationary phase in which the cells persist for at least 40 h prior to a slower lysis trend. Data are representative of three replications (n = 3). Error bars represent the standard deviation of biological replicates.
