# Supplementary material for: Growth of Porphyromonas gingivalis on human serum albumin triggers programmed cell death
Source: J Oral Microbiol. 2022 Dec 22;15(1):2161182. doi: 10.1080/20002297.2022.2161182 (PMC9788703; doi:10.1080/20002297.2022.2161182)
Supplement: Supplemental Material [file ZJOM_A_2161182_SM7719.zip › supplementary files/HSA_Figures Supplemental S2.pdf]

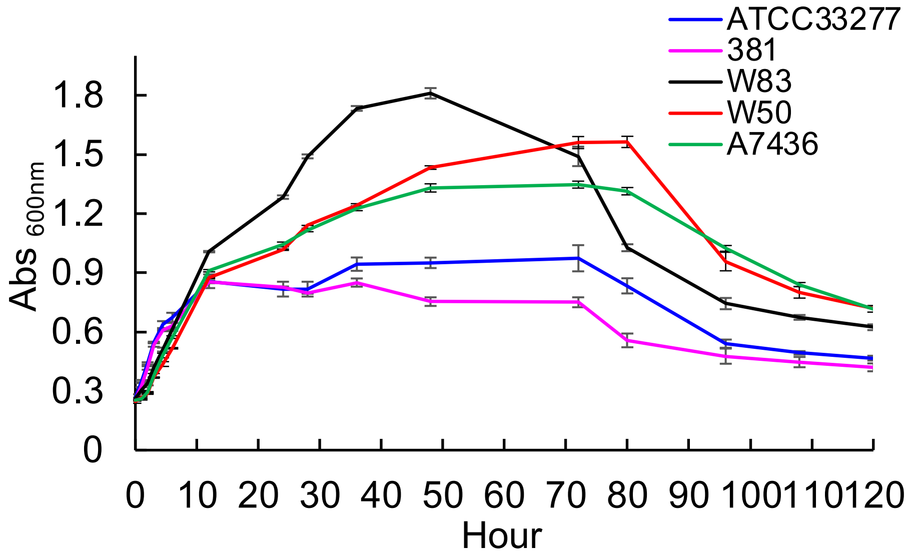

Figure S2. Growth rate of *P. gingivalis* strains W50, W83, 381, ATCC33277, and A7436 in 1% BSAHK medium. W83 grew exponentially for about 50 h to a maximum OD_{600} of 1.81 followed by a very short stationary phase leading to a gradual cell lysis rate. 381 and ATCC 33277 stopped logarithmic growth at 12 h after incubation, with the maximum OD600 of 0.91; these strains survived in stationary phase for up to 70 h followed by a slower lysis rate. W50 and A7436 displayed a relatively similar exponential growth rate, growing much slower than W83. W50 continued its logarithmic growth for up to 80 h of incubation followed by a short stationary phase and a gradual cell lysis. Data are representative of three replications (n = 3). Error bars represent the standard deviation of biological replicates.
