# Supplementary material for: Growth of Porphyromonas gingivalis on human serum albumin triggers programmed cell death
Source: J Oral Microbiol. 2022 Dec 22;15(1):2161182. doi: 10.1080/20002297.2022.2161182 (PMC9788703; doi:10.1080/20002297.2022.2161182)
Supplement: Supplemental Material [file ZJOM_A_2161182_SM7719.zip › supplementary files/HSA_Figures Supplemental S3.pdf]

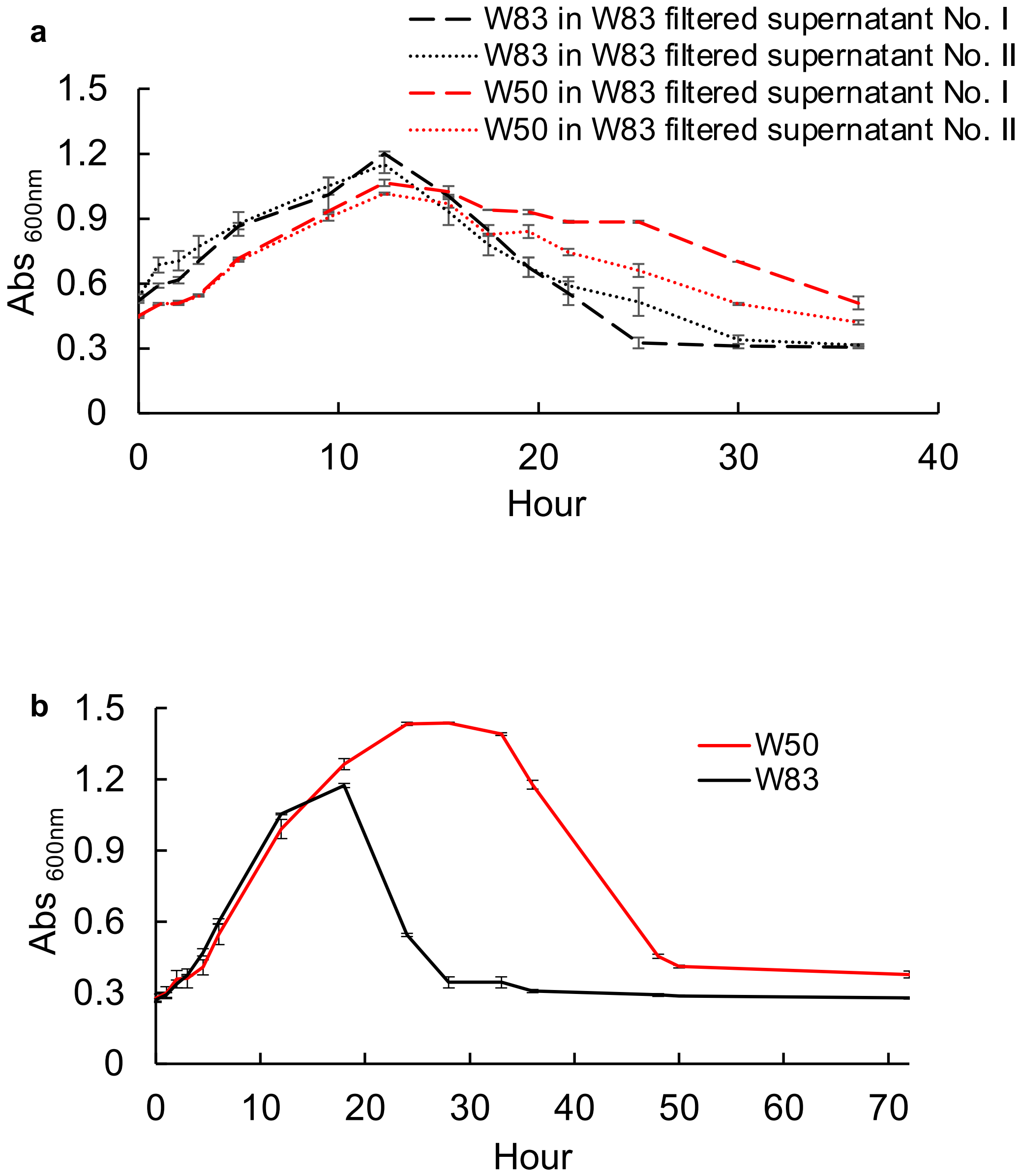

Figure S3. Growth rate of *P. gingivalis* strains W50 and W83 in the cell-free supernatant of W83 at early (No. I) and lysis (No. II) phases in 1% HSAHK medium. Both active cells with OD_{600} of ~ 0.5 of W83 and W50 sustained exponential growth and viability for at least 12 h after addition of the lysis supernatant (**a**), and graph b shows the growth rate of W83 and W50 without adding the cell-free supernatant of W83 as a control (**b**), These results indicate neither extracellular lytic byproducts nor nutrient depletion underlies the fast lysis event. Data are representative of three replications (n = 3). Error bars represent the standard deviation of biological replicates.
