# Supplementary material for: Growth of Porphyromonas gingivalis on human serum albumin triggers programmed cell death
Source: J Oral Microbiol. 2022 Dec 22;15(1):2161182. doi: 10.1080/20002297.2022.2161182 (PMC9788703; doi:10.1080/20002297.2022.2161182)
Supplement: Supplemental Material [file ZJOM_A_2161182_SM7719.zip › supplementary files/HSA_Figures Supplemental S4.pdf]

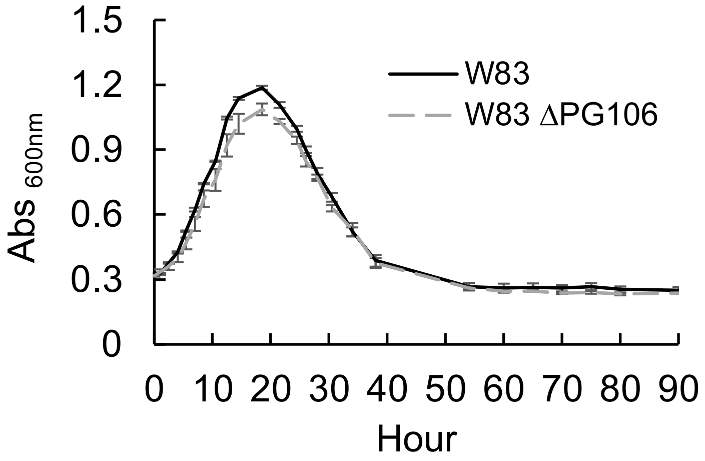

Figure S4. Assessment of growth rate of *P. gingivalis* strains W83 and W83 ΔPG0106 (capsule null strain) in 1% HSAHK medium. W83 ΔPG0106 was similar to the parental strain, indicating heterogeneity in growth and lysis rate is not dependent on the cell encapsulation. Data are representative of three replications (n = 3). Error bars represent the standard deviation of technical replicates.
