# Supplementary material for: Growth of Porphyromonas gingivalis on human serum albumin triggers programmed cell death
Source: J Oral Microbiol. 2022 Dec 22;15(1):2161182. doi: 10.1080/20002297.2022.2161182 (PMC9788703; doi:10.1080/20002297.2022.2161182)
Supplement: Supplemental Material [file ZJOM_A_2161182_SM7719.zip › supplementary files/HSA_Figures Supplemental S5.pdf]

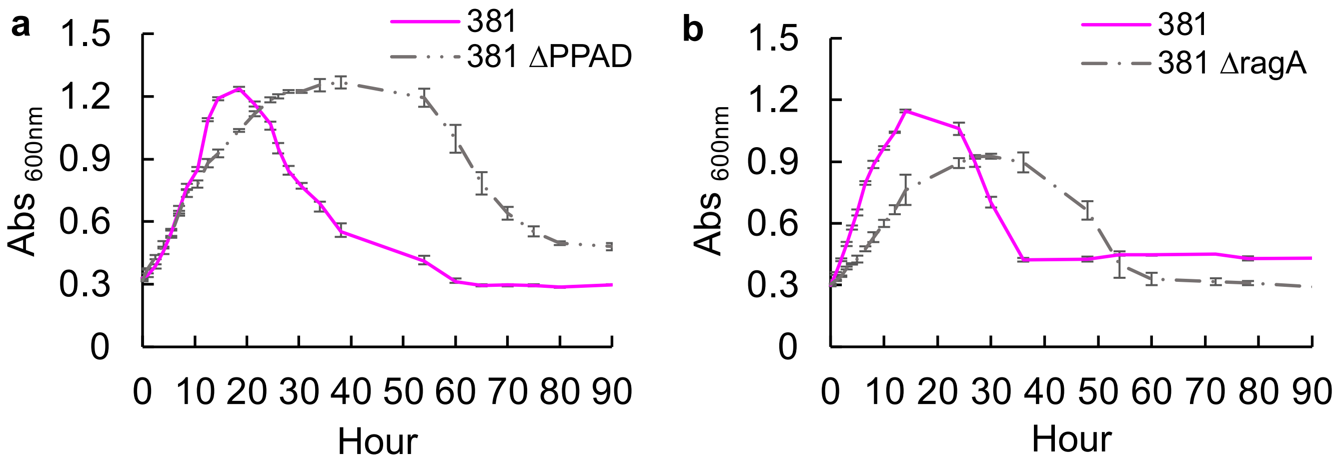

Figure S5. Assessment of growth rate of *P. gingivalis* mutants in 1% HSAHK medium (**a**) Growth rate of 381 and 381 Δ*ppad*, and (**b**) growth rate of 381 and 381 Δ*ragA*. Both mutants displayed a slower logarithmic growth, significant delay in transition to the stationary phase, and longer survival in stationary phase when compared with the parental strain. Data are representative of three replications (n = 3). Error bars represent the standard deviation of technical replicates.
