# Supplementary material for: Growth of Porphyromonas gingivalis on human serum albumin triggers programmed cell death
Source: J Oral Microbiol. 2022 Dec 22;15(1):2161182. doi: 10.1080/20002297.2022.2161182 (PMC9788703; doi:10.1080/20002297.2022.2161182)
Supplement: Supplemental Material [file ZJOM_A_2161182_SM7719.zip › supplementary files/HSA_Figures Supplemental S6.pdf]

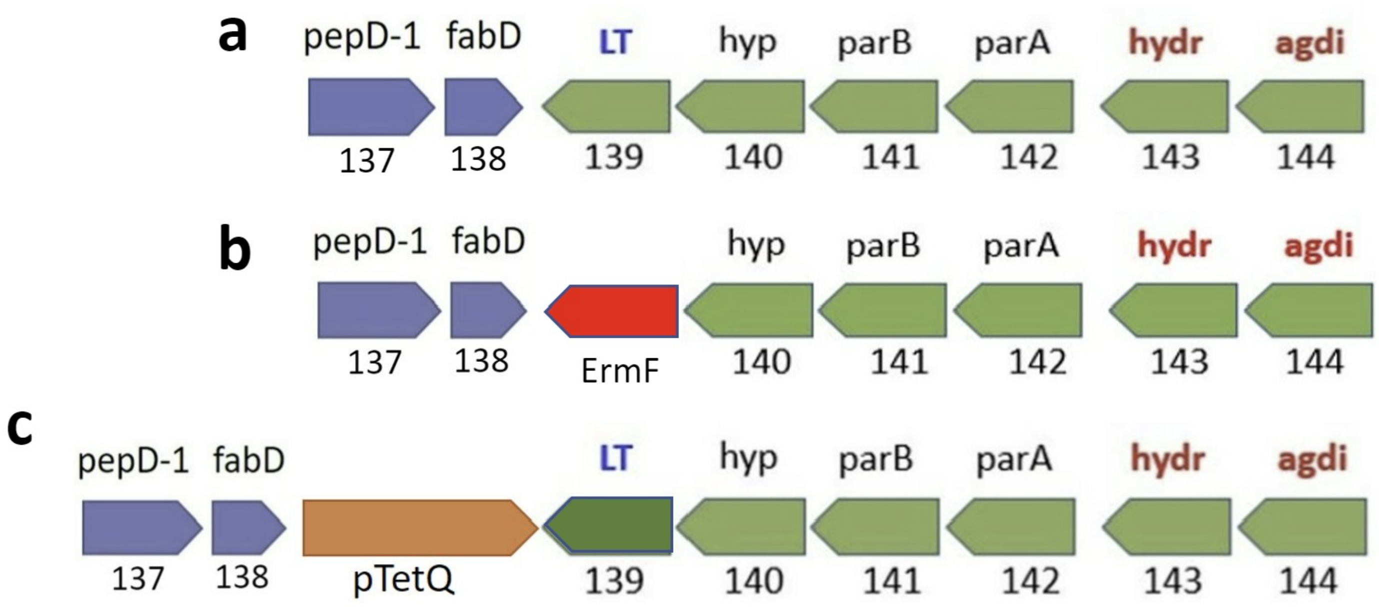

Genomic context of PG0139 within chromosomal region shown to be upregulated in strain W83 by RNA-Seq and diagrams of deletion and complementation designs. (a) Operon prediction and genomic context in strain W83 and W50. (b) Deletion of PG0139 and replacement of an in-frame promoterless erythromycin resistance cassette. (c) Complementation by knock-in of PG0139 and the addition of tetracycline resistance cassette as an antibiotic selection marker.
