# Supplementary material for: Growth of Porphyromonas gingivalis on human serum albumin triggers programmed cell death
Source: J Oral Microbiol. 2022 Dec 22;15(1):2161182. doi: 10.1080/20002297.2022.2161182 (PMC9788703; doi:10.1080/20002297.2022.2161182)
Supplement: Supplemental Material [file ZJOM_A_2161182_SM7719.zip › supplementary files/Supplemental Table S1a.docx]

**Supplemental Table S1a.** Differential gene expression analyzed by pairwise comparison of the transcriptomes of strain W83 and W50 at 12.5hrs (exponential phase of growth). Gene number and predicted function are provided. Highlighted in green are genes that are discussed in the manuscript. (Fold change ≥ 2; *q*-value < 0.01)

| **Name** | **Old Name** | **Annotation** | **logFC** |
| --- | --- | --- | --- |
| PG_RS00900 | PG0195 | rubrerythrin family protein | 6.61 |
| PG_RS02400 | PG0537 | aminoacyl-histidine dipeptidase | 5.91 |
| PG_RS02770 | PG0627 | RNA-binding protein | 5.70 |
| PG_RS07970 | PG1808 | bifunctional (p)ppGpp synthetase/ hydrolase | 4.78 |
| PG_RS07925 | PG1797 | response regulator | 4.68 |
| PG_RS00250 | PG0055 | PspC domain-containing protein | 4.67 |
| PG_RS05630 | PG1277 | nucleotide sugar dehydrogenase | 4.67 |
| PG_RS00205 | PG0043 | family 20 glycosylhydrolase | 4.58 |
| PG_RS07820 | PG1777 | DUF59 domain-containing protein | 4.48 |
| PG_RS09865 | PG2219 | TrkH family potassium uptake protein | 4.39 |
| polA | PG1794 | DNA polymerase I | 4.37 |
| PG_RS05185 | PG1171 | (Fe-S)-binding protein | 4.25 |
| PG_RS06465 | PG1471 | PH domain-containing protein | 4.25 |
| PG_RS02655 | PG0598 | YjgP/YjgQ family permease | 4.25 |
| PG_RS09350 | PG2109 | thiamine phosphate synthase | 4.22 |
| PG_RS00785 | PG0171 | bifunctional metallophosphatase/5'-nucleotidase | 4.22 |
| PG_RS09395 | PG2121 | asparaginase | 4.21 |
| PG_RS09565 | PG2157 | M28 family peptidase | 4.14 |
| pssA | PG0964 | CDP-diacylglycerol--serine O-phosphatidyltransferase | 4.12 |
| murD | PG0578 | UDP-N-acetylmuramoyl-L-alanine--D-glutamate ligase | 4.12 |
| PG_RS04165 | PG0945 | ABC transporter permease | 4.12 |
| PG_RS00285 | PG0062 | AAA family ATPase | 3.97 |
| PG_RS08985 | PG2034 | sulfide/dihydroorotate dehydrogenase-like FAD/NAD-binding protein | 3.97 |
| PG_RS06185 | PG1405 | LPS-assembly protein LptD | 3.97 |
| clpB | PG1118 | ATP-dependent chaperone ClpB | 3.84 |
| PG_RS04935 | PG1114 | aspartate 1-decarboxylase | 3.82 |
| kdsA | PG1743 | 3-deoxy-8-phosphooctulonate synthase | 3.82 |
| PG_RS01395 | PG0311 | glycosyltransferase family 2 protein | 3.82 |
| PG_RS08095 | PG1841 | DUF2156 domain-containing protein | 3.82 |
| PG_RS05045 | PG1136 | asparagine synthetase B family protein | 3.82 |
| scpA | PG1657 | methylmalonyl-CoA mutase | 3.75 |
| PG_RS00095 | PG0020 | winged helix-turn-helix transcriptional regulator | 3.64 |
| PG_RS00670 | PG0144 | agmatine deiminase family protein | 3.64 |
| PG_RS10480 | PG1421 | 4Fe-4S binding protein | 3.59 |
| PG_RS03395 | PG0776 | electron transfer flavoprotein subunit alpha/FixB | 3.39 |
| PG_RS07395 | PG1677 | phosphoglycerate kinase | 3.30 |
| rnr | PG1721 | ribonuclease R | 3.09 |
| PG_RS02010 | PG0451 | HlyC/CorC family transporter | 3.00 |
| PG_RS07480 | PG1697 | DEAD/DEAH box helicase family protein | 2.98 |
| ssb | PG0271 | single-stranded DNA-binding protein | 2.93 |
| PG_RS07255 | PG1648 | bifunctional (p)ppGpp synthetase/ hydrolase | 2.92 |
| PG_RS05945 | PG1351 | hypothetical protein | 2.90 |
| PG_RS07125 | PG1616 | succinate dehydrogenase/fumarate reductase cytochrome b subunit | 2.89 |
| PG_RS03400 | PG0777 | electron transfer flavoprotein subunit beta/FixA family protein | 2.86 |
| PG_RS07980 | PG1810 | 2-oxoglutarate oxidoreductase | 2.86 |
| mutA | PG1656 | methylmalonyl-CoA mutase small subunit | 2.85 |
| PG_RS00630 | PG0136 | flippase-like domain-containing protein | 2.84 |
| PG_RS01910 | PG0429 | 2-oxoacid:acceptor oxidoreductase subunit alpha | 2.82 |
| PG_RS07990 | PG1813 | 4Fe-4S dicluster domain-containing protein | 2.79 |
| PG_RS07120 | PG1615 | fumarate reductase/succinate dehydrogenase flavoprotein | 2.66 |
| PG_RS08925 | PG2022 | membrane protein | 2.65 |
| PG_RS07710 | PG1754 | S9 family peptidase | 2.65 |
| PG_RS09040 | PG2047 | AAA family ATPase | 2.64 |
| PG_RS03210 | PG0733 | riboflavin synthase | 2.64 |
| msrB | PG2088 | peptide-methionine (R)-S-oxide reductase MsrB | 2.64 |
| PG_RS08235 | PG1878 | cysteine--tRNA ligase | 2.63 |
| PG_RS10650 |  | hypothetical protein | 2.59 |
| PG_RS07905 | PG1792 | sodium:hydrogen antiporter | 2.58 |
| PG_RS07410 | PG1681 | 4-alpha-glucanotransferase | 2.55 |
| PG_RS09305 | PG2099 | DEAD/DEAH box helicase | 2.54 |
| PG_RS00665 | PG0143 | carbon-nitrogen hydrolase | 2.53 |
| PG_RS06745 | PG1530 | GTP pyrophosphokinase | 2.53 |
| megL | PG0343 | methionine gamma-lyase | 2.52 |
| PG_RS02945 | PG0670 | ABC transporter substrate-binding protein | 2.52 |
| PG_RS07975 | PG1809 | 2-oxoglutarate ferredoxin oxidoreductase subunit gamma | 2.49 |
| PG_RS07985 | PG1812 | 3-methyl-2-oxobutanoate dehydrogenase subunit VorB | 2.45 |
| rsmH | PG0573 | 16S rRNA (cytosine(1402)-N(4))-methyltransferase RsmH | 2.44 |
| PG_RS03440 | PG0784 | polyprenyl synthetase family protein | 2.41 |
| PG_RS03760 | PG0854 | DUF4393 domain-containing protein | 2.41 |
| PG_RS07150 | PG1622 | DNA gyrase/topoisomerase IV subunit A | 2.41 |
| PG_RS07835 | PG1780 | Serine palmitoyl-transferase | 2.32 |
| PG_RS01235 | PG0276 | hypothetical protein | 2.31 |
| PG_RS06220 | PG1414 | TonB-dependent receptor plug domain-containing protein | 2.28 |
| PG_RS07070 | PG1604 | hypothetical protein | 2.28 |
| PG_RS07030 | PG1596 | isoleucine--tRNA ligase | 2.26 |
| nrfH | PG1821 | cytochrome c nitrite reductase small subunit | 2.22 |
| PG_RS01445 | PG0322 | dicarboxylate/amino acid:cation symporter | 2.20 |
| PG_RS07205 | PG1636 | DNA translocase FtsK | 2.14 |
| dxs | PG2217 | 1-deoxy-D-xylulose-5-phosphate synthase | 2.14 |
| PG_RS08650 | PG1960 | 50S ribosomal protein L28 | 2.11 |
| PG_RS05805 | PG1318 | sigma-70 family RNA polymerase sigma factor | 2.08 |
| PG_RS07415 | PG1682 | glycosyltransferase | 2.08 |
| pdxB | PG1220 | 4-phosphoerythronate dehydrogenase PdxB | 2.05 |
| nrfA | PG1820 | ammonia-forming cytochrome c nitrite reductase | 2.03 |
| PG_RS08615 | PG1951 | glutamine--tRNA ligase/YqeY domain fusion protein | 2.02 |
| PG_RS08240 | PG1879 | patatin-like phospholipase family protein | 2.02 |
| PG_RS04385 | PG0991 | translation initiation factor IF-3 | 2.00 |
| PG_RS07595 | PG1726 | PDZ domain-containing protein | 1.99 |
| PG_RS05965 | PG1355 | acyltransferase | 1.96 |
| gmd | PG1288 | GDP-mannose 4 2C6-dehydratase | 1.96 |
| PG_RS05650 | PG1281 | DUF2027 domain-containing protein | 1.94 |
| PG_RS01575 | PG0355 | hypothetical protein | 1.88 |
| PG_RS01025 | PG0224 | MarC family protein | 1.86 |
| nusA | PG0254 | transcription termination/antitermination protein NusA | 1.84 |
| bamD | PG1215 | outer membrane protein assembly factor BamD | 1.83 |
| PG_RS03390 | PG0775 | acyl-CoA dehydrogenase | 1.82 |
| PG_RS07685 | PG1748 | transketolase | 1.80 |
| PG_RS01915 | PG0430 | 2-oxoacid:ferredoxin oxidoreductase subunit beta | 1.79 |
| PG_RS05540 | PG1258 | integration host factor subunit beta | 1.79 |
| PG_RS04310 | PG0975 | PhoH family protein | 1.78 |
| PG_RS05085 | PG1143 | UDP-glucose/GDP-mannose dehydrogenase family protein | 1.78 |
| PG_RS07115 | PG1614 | succinate dehydrogenase/fumarate reductase iron-sulfur | 1.76 |
| thrS | PG0992 | threonine--tRNA ligase | 1.76 |
| lysA | PG2188 | diaminopimelate decarboxylase | 1.74 |
| PG_RS01135 | PG0249 | oxaloacetate decarboxylase | 1.73 |
| PG_RS02700 | PG0611 | hypothetical protein | 1.72 |
| PG_RS09610 | PG2168 | DUF3575 domain-containing protein | 1.71 |
| PG_RS01390 | PG0310 | nitroreductase | 1.70 |
| PG_RS07475 | PG1696 | hypothetical protein | 1.66 |
| frr | PG1901 | ribosome recycling factor | 1.66 |
| PG_RS00425 | PG0094 | TolC family protein | 1.65 |
| PG_RS03435 | PG0783 | TatD family hydrolase | 1.65 |
| PG_RS02555 | PG0577 | phospho-N-acetylmuramoyl-pentapeptide-transferase | 1.65 |
| PG_RS09135 | PG2066 | DUF4837 family protein | 1.65 |
| PG_RS07310 | PG1663 | ABC transporter ATP-binding protein | 1.65 |
| PG_RS05445 | PG1238 | RluA family pseudouridine synthase | 1.64 |
| PG_RS05970 | PG1356 | hypothetical protein | 1.61 |
| PG_RS07420 | PG1683 | alpha-amylase | 1.59 |
| gldE | PG0272 | gliding motility-associated protein GldE | 1.59 |
| PG_RS08090 | PG1837 | DUF2436 domain-containing protein | 1.58 |
| meaB | PG0321 | methylmalonyl Co-A mutase-associated GTPase MeaB | 1.57 |
| pckA | PG1676 | phosphoenolpyruvate carboxykinase (ATP) | 1.57 |
| dnaJ | PG1776 | molecular chaperone DnaJ | 1.57 |
| dnaA | PG0001 | chromosomal replication initiator protein DnaA | 1.53 |
| PG_RS05430 | PG1235 | NAD-dependent epimerase/dehydratase family protein | 1.53 |
| PG_RS05820 | PG1323 | PhoH family protein | 1.53 |
| mscL | PG1330 | large-conductance mechanosensitive channel protein MscL | 1.51 |
| PG_RS00950 | PG0209 | formate/nitrite transporter family protein | 1.51 |
| PG_RS00200 | PG0042 | serine hydroxymethyltransferase | 1.50 |
| PG_RS08970 | PG2031 | hypothetical protein | 1.47 |
| PG_RS08655 | PG1961 | phosphate transporter family protein | 1.47 |
| PG_RS05955 | PG1353 | orotate phosphoribosyltransferase | 1.47 |
| PG_RS04650 | PG1056 | 6-carboxytetrahydropterin synthase | 1.43 |
| PG_RS06975 | PG1585 | protein BatD | 1.42 |
| ffh | PG1115 | signal recognition particle protein | 1.41 |
| PG_RS02080 | PG0468 | mannose-6-phosphate isomerase | 1.40 |
| PG_RS09335 | PG2106 | PorT family protein | 1.40 |
| PG_RS01560 | PG0350 | hypothetical protein | 1.35 |
| PG_RS03355 | PG0766 | polyribonucleotide nucleotidyltransferase | 1.32 |
| PG_RS00635 | PG0137 | aminoacyl-histidine dipeptidase | 1.32 |
| PG_RS04250 | PG0962 | proline--tRNA ligase | 1.30 |
| PG_RS07105 | PG1612 | acyl-CoA carboxylase subunit beta | 1.30 |
| galK | PG1633 | galactokinase | 1.29 |
| PG_RS00445 | PG0099 | phenylalanine--tRNA ligase subunit beta | 1.29 |
| PG_RS03330 | PG0758 | M3 family metallopeptidase | 1.28 |
| PG_RS07660 | PG1741 | aspartate ammonia-lyase | 1.28 |
| PG_RS05260 | PG1189 | DUF349 domain-containing protein | 1.28 |
| PG_RS01435 | PG0320 | DUF1573 domain-containing protein | 1.27 |
| dnaK | PG1208 | molecular chaperone DnaK | 1.25 |
| PG_RS09725 | PG2190 | ATP-binding cassette domain-containing protein | 1.24 |
| PG_RS07485 | PG1701 | gamma-glutamyl-gamma-aminobutyrate hydrolase family protein | 1.24 |
| PG_RS01510 | PG0336 | hypothetical protein | 1.24 |
| PG_RS01800 | PG0404 | hypothetical protein | 1.22 |
| PG_RS09295 | PG2096 | AsmA family protein | 1.22 |
| rny | PG0401 | ribonuclease Y | 1.21 |
| PG_RS09795 | PG2207 | NAD(P)-dependent oxidoreductase | 1.21 |
| fabD | PG0138 | ACP S-malonyltransferase | 1.19 |
| PG_RS08280 | PG1889 | hypothetical protein | 1.19 |
| PG_RS09720 | PG2189 | aspartate kinase | 1.18 |
| murB | PG1342 | UDP-N-acetylmuramate dehydrogenase | 1.17 |
| PG_RS07085 | PG1608 | sodium ion-translocating decarboxylase subunit beta | 1.17 |
| mce | PG1613 | methylmalonyl-CoA epimerase | 1.17 |
| PG_RS06455 | PG1469 | N-6 DNA methylase | 1.14 |
| PG_RS06300 | PG1435 | site-specific integrase | 1.14 |
| nifJ | PG0548 | pyruvate:ferredoxin (flavodoxin) oxidoreductase | 1.13 |
| PG_RS09840 | PG2214 | DUF2851 family protein | 1.10 |
| nqrA | PG2182 | NADH:ubiquinone reductase (Na(+)-transporting) subunit A | 1.10 |
| PG_RS02575 | PG0581 | UDP-N-acetylmuramate--L-alanine ligase | 1.09 |
| PG_RS04700 | PG1068 | 3-keto-5-aminohexanoate cleavage protein | 1.08 |
| PG_RS08600 | PG1949 | malate dehydrogenase | 1.06 |
| dnaN | PG1853 | DNA polymerase III subunit beta | 1.05 |
| infB | PG0255 | translation initiation factor IF-2 | 1.05 |
| PG_RS05330 | PG1210 | aminopeptidase P family protein | 1.05 |
| PG_RS03445 | PG0785 | energy transducer TonB | 1.04 |
| PG_RS02195 | PG0495 | T9SS type A sorting domain-containing protein | 1.04 |
| PG_RS03480 | PG0793 | fructose-bisphosphatase class III | 1.01 |
| PG_RS04235 | PG0959 | Mrp/NBP35 family ATP-binding protein | 1.01 |
| trxA | PG0034 | thioredoxin | -1.02 |
| PG_RS08255 | PG1884 | alpha-L-fucosidase | -1.03 |
| PG_RS06820 | PG1545 | superoxide dismutase | -1.07 |
| PG_RS05815 | PG1321 | formate--tetrahydrofolate ligase | -1.08 |
| tpx | PG1729 | thiol peroxidase | -1.10 |
| PG_RS03105 | PG0707 | TonB-dependent receptor | -1.11 |
| PG_RS08590 | PG1947 | tetratricopeptide repeat protein | -1.11 |
| PG_RS09785 | PG2205 | 2-dehydropantoate 2-reductase | -1.12 |
| PG_RS11175 |  | hypothetical protein | -1.12 |
| PG_RS06850 | PG1553 | cobaltochelatase subunit CobN | -1.13 |
| PG_RS02185 | PG0491 | S46 family peptidase | -1.15 |
| PG_RS01665 | PG0378 | elongation factor Ts | -1.15 |
| PG_RS02480 | PG0558 | purine nucleoside phosphorylase I 2C inosine and guanosine-specific | -1.17 |
| rplL | PG0393 | 50S ribosomal protein L7/L12 | -1.17 |
| PG_RS06055 | PG1374 | T9SS type A sorting domain-containing protein | -1.17 |
| PG_RS03290 | PG0750 | glycosyltransferase | -1.18 |
| rpoC | PG0395 | DNA-directed RNA polymerase subunit beta' | -1.19 |
| PG_RS00890 | PG0193 | OmpH family outer membrane protein | -1.20 |
| sufC | PG0258 | Fe-S cluster assembly ATPase SufC | -1.22 |
| rpoB | PG0394 | DNA-directed RNA polymerase subunit beta | -1.25 |
| PG_RS00110 | PG0024 | redox-sensing transcriptional repressor Rex | -1.25 |
| deoC | PG1996 | deoxyribose-phosphate aldolase | -1.26 |
| PG_RS01230 | PG0275 | redoxin domain-containing protein | -1.35 |
| porU | PG0026 | T9SS sortase PorU | -1.35 |
| PG_RS05670 | PG1286 | ferritin | -1.36 |
| PG_RS00115 | PG0025 | fumarylacetoacetate hydrolase family protein | -1.41 |
| PG_RS07965 | PG1807 | ATP synthase subunit C | -1.44 |
| PG_RS00790 | PG0172 | 3'-5' exonuclease | -1.45 |
| PG_RS02630 | PG0593 | DegQ family serine endoprotease | -1.45 |
| PG_RS06840 | PG1551 | heme-binding protein HmuY | -1.46 |
| PG_RS04065 | PG0925 | thymidine kinase | -1.47 |
| PG_RS08055 | PG1827 | RNA polymerase sigma factor | -1.50 |
| PG_RS09835 | PG2213 | (2Fe-2S)-binding protein | -1.51 |
| PG_RS04800 | PG1089 | response regulator transcription factor | -1.56 |
| PG_RS00405 | PG0090 | DNA starvation/stationary phase protection protein | -1.70 |
| PG_RS01310 |  | chromate transporter | -1.73 |
| PG_RS08595 | PG1948 | alpha/beta hydrolase | -1.74 |
| PG_RS01450 | PG0323 | cupin domain-containing protein | -1.76 |
| queA | PG1540 | tRNA preQ1(34) S-adenosylmethionine ribosyltransferase-isomerase QueA | -1.82 |
| PG_RS09765 | PG2200 | tetratricopeptide repeat protein | -1.83 |
| secA | PG0514 | preprotein translocase subunit SecA | -1.85 |
| PG_RS04080 | PG0928 | PglZ domain-containing protein | -1.87 |
| hflB | PG0047 | ATP-dependent metallopeptidase FtsH/Yme1/Tma | -1.88 |
| PG_RS02055 | PG0462 | MFS transporter | -1.99 |
| PG_RS02410 | PG0539 | efflux RND transporter periplasmic adaptor subunit | -2.08 |
| tsaE | PG0927 | tRNA (adenosine(37)-N6)-threonylcarbamoyltransferase complex ATPase subunit type 1 TsaE | -2.34 |
| ahpC | PG0618 | peroxiredoxin | -2.35 |
| PG_RS07930 | PG1798 | T9SS type A sorting domain-containing protein | -2.43 |
| PG_RS08170 | PG1858 | flavodoxin | -2.47 |
| PG_RS08165 | PG1857 | DUF2023 family protein | -2.50 |
| PG_RS07950 | PG1804 | V-type ATP synthase subunit B | -2.61 |
| rgpB | PG0506 | Arg-gingipain RgpB | -2.64 |
| PG_RS03145 | PG0717 | hypothetical protein | -3.08 |
| PG_RS07170 | PG1625 | hypothetical protein | -3.68 |
| PG_RS04980 | PG1124 | cob(I)yrinic acid a 2Cc-diamide adenosyltransferase | -3.80 |
| PG_RS02420 | PG0541 | hypothetical protein | -3.88 |
| ahpF | PG0619 | alkyl hydroperoxide reductase subunit F | -3.96 |
| PG_RS01870 | PG0421 | DUF2807 domain-containing protein | -4.24 |
| PG_RS07175 | PG1626 | transporter | -4.41 |
| PG_RS03020 | PG0686 | DUF1858 domain-containing protein | -5.10 |
| PG_RS02415 | PG0540 | efflux RND transporter permease subunit | -5.10 |
| PG_RS11080 |  | DUF1661 domain-containing protein | -6.68 |
