# Supplementary material for: Growth of Porphyromonas gingivalis on human serum albumin triggers programmed cell death
Source: J Oral Microbiol. 2022 Dec 22;15(1):2161182. doi: 10.1080/20002297.2022.2161182 (PMC9788703; doi:10.1080/20002297.2022.2161182)
Supplement: Supplemental Material [file ZJOM_A_2161182_SM7719.zip › supplementary files/Supplemental Table S1b.docx]

**Supplemental Table S1b.** Differential gene expression analyzed by pairwise comparison of the transcriptomes of strain **W83 (12.5hrs) and W50 (20 hrs)**; exponential phase of growth. Gene number and predicted function are provided. Highlighted in green are genes that are discussed in the manuscript. (Fold change ≥ 2; *q*-value < 0.01)

| Name | oldName | Annotation | logFC |
| --- | --- | --- | --- |
| PG_RS01910 | PG0429 | 2-oxoacid:acceptor oxidoreductase subunit alpha | 6.90 |
| PG_RS02195 | PG0495 | T9SS type A sorting domain-containing protein | 5.96 |
| PG_RS02400 | PG0537 | aminoacyl-histidine dipeptidase | 5.59 |
| PG_RS07835 | PG1780 | Serine palmitoyltransferase (SPT) | 5.50 |
| PG_RS07395 | PG1677 | phosphoglycerate kinase | 5.19 |
| PG_RS00950 | PG0209 | formate/nitrite transporter family protein | 5.06 |
| PG_RS07970 | PG1808 | bifunctional (p)ppGpp synthetase/ hydrolase | 4.84 |
| PG_RS00630 | PG0136 | flippase-like domain-containing protein | 4.73 |
| mutA | PG1656 | methylmalonyl-CoA mutase small subunit | 4.72 |
| PG_RS00900 | PG0195 | rubrerythrin family protein | 4.58 |
| PG_RS01025 | PG0224 | MarC family protein | 4.53 |
| PG_RS02945 | PG0670 | ABC transporter substrate-binding protein | 4.40 |
| PG_RS00745 | PG0160 | hypothetical protein | 4.33 |
| PG_RS06465 | PG1471 | PH domain-containing protein | 4.30 |
| PG_RS07000 | PG1591 | type I restriction-modification system subunit R | 4.30 |
| PG_RS02625 | PG0592 | type B 50S ribosomal protein L31 | 4.02 |
| PG_RS02770 | PG0627 | RNA-binding protein | 3.98 |
| PG_RS03400 | PG0777 | electron transfer flavoprotein subunit beta/FixA family protein | 3.97 |
| PG_RS01395 | PG0311 | glycosyltransferase family 2 protein | 3.87 |
| PG_RS08095 | PG1841 | DUF2156 domain-containing protein | 3.87 |
| PG_RS05045 | PG1136 | asparagine synthetase B family protein | 3.87 |
| PG_RS05805 | PG1318 | sigma-70 family RNA polymerase sigma factor | 3.86 |
| cas8c | PG2018 | type I-PGING CRISPR-associated protein Cas8c/Csp2 | 3.85 |
| mscL | PG1330 | large-conductance mechanosensitive channel protein MscL | 3.83 |
| PG_RS00670 | PG0144 | agmatine deiminase family protein | 3.69 |
| PG_RS00095 | PG0020 | winged helix-turn-helix transcriptional regulator | 3.69 |
| PG_RS09235 | PG2083 | DUF4831 family protein | 3.69 |
| tgt | PG0500 | tRNA guanosine(34) transglycosylase Tgt | 3.69 |
| kdsB | PG1815 | 3-deoxy-manno-octulosonate cytidylyltransferase | 3.69 |
| PG_RS00390 | PG0087 | SIS domain-containing protein | 3.67 |
| PG_RS01915 | PG0430 | 2-oxoacid:ferredoxin oxidoreductase subunit beta | 3.67 |
| PG_RS04720 | PG1072 | DNA mismatch repair protein MutS | 3.58 |
| PG_RS06270 | PG1430 | tetratricopeptide repeat protein | 3.55 |
| PG_RS09610 | PG2168 | DUF3575 domain-containing protein | 3.47 |
| PG_RS07040 | PG1598 | lipoprotein signal peptidase | 3.33 |
| PG_RS09555 | PG2155 | TraB/GumN family protein | 3.30 |
| PG_RS03395 | PG0776 | electron transfer flavoprotein subunit alpha/FixB family protein | 3.29 |
| PG_RS10480 | PG1421 | 4Fe-4S binding protein | 3.16 |
| thrS | PG0992 | threonine--tRNA ligase | 3.12 |
| gltA | PG2033 | NADPH-dependent glutamate synthase | 3.12 |
| PG_RS05430 | PG1235 | NAD-dependent epimerase/dehydratase family protein | 3.05 |
| PG_RS02935 | PG0668 | TonB-dependent receptor | 2.93 |
| PG_RS06455 | PG1469 | N-6 DNA methylase | 2.88 |
| PG_RS01560 | PG0350 | hypothetical protein | 2.87 |
| PG_RS07575 | PG1723 | 30S ribosomal protein S20 | 2.85 |
| PG_RS07935 | PG1801 | hypothetical protein | 2.76 |
| PG_RS08925 | PG2022 | membrane protein | 2.76 |
| PG_RS03435 | PG0783 | TatD family hydrolase | 2.66 |
| PG_RS06745 | PG1530 | GTP pyrophosphokinase | 2.65 |
| rpsG | PG1941 | 30S ribosomal protein S7 | 2.63 |
| PG_RS03390 | PG0775 | acyl-CoA dehydrogenase | 2.62 |
| PG_RS08650 | PG1960 | 50S ribosomal protein L28 | 2.54 |
| PG_RS05980 | PG1358 | GNAT family N-acetyltransferase | 2.53 |
| PG_RS09165 | PG2072 | UvrD-helicase domain-containing protein | 2.45 |
| PG_RS05165 | PG1164 | hypothetical protein | 2.40 |
| PG_RS07070 | PG1604 | hypothetical protein | 2.40 |
| PG_RS05260 | PG1189 | DUF349 domain-containing protein | 2.38 |
| PG_RS02700 | PG0611 | hypothetical protein | 2.36 |
| PG_RS04385 | PG0991 | translation initiation factor IF-3 | 2.35 |
| PG_RS01445 | PG0322 | dicarboxylate/amino acid:cation symporter | 2.34 |
| PG_RS08280 | PG1889 | hypothetical protein | 2.29 |
| PG_RS07205 | PG1636 | DNA translocase FtsK | 2.27 |
| ssb | PG0271 | single-stranded DNA-binding protein | 2.27 |
| PG_RS10650 |  | hypothetical protein | 2.26 |
| PG_RS04995 | PG1127 | winged helix-turn-helix transcriptional regulator | 2.26 |
| PG_RS04310 | PG0975 | PhoH family protein | 2.25 |
| PG_RS07255 | PG1648 | bifunctional (p)ppGpp synthetase/ hydrolase | 2.24 |
| PG_RS08655 | PG1961 | phosphate transporter family protein | 2.24 |
| PG_RS07030 | PG1596 | isoleucine--tRNA ligase | 2.23 |
| rpsM | PG1914 | 30S ribosomal protein S13 | 2.22 |
| PG_RS09655 | PG2175 | TlpA family protein disulfide reductase | 2.17 |
| PG_RS00905 | PG0196 | insulinase family protein | 2.16 |
| PG_RS07990 | PG1813 | 4Fe-4S dicluster domain-containing protein | 2.14 |
| PG_RS05085 | PG1143 | UDP-glucose/GDP-mannose dehydrogenase family protein | 2.11 |
| PG_RS02440 | PG0547 | ATP-binding protein | 2.10 |
| PG_RS02745 | PG0622 | DoxX family protein | 2.09 |
| PG_RS00425 | PG0094 | TolC family protein | 2.08 |
| PG_RS09485 | PG2139 | DUF177 domain-containing protein | 2.08 |
| PG_RS07125 | PG1616 | succinate dehydrogenase/fumarate reductase cytochrome b | 2.06 |
| PG_RS04535 | PG1030 | T9SS type A sorting domain-containing protein | 2.01 |
| mce | PG1613 | methylmalonyl-CoA epimerase | 2.00 |
| PG_RS06255 | PG1424 | peptidylarginine deiminase PPAD | 1.99 |
| PG_RS08970 | PG2031 | hypothetical protein | 1.97 |
| PG_RS09685 | PG2180 | Na(+)-translocating NADH-quinone reductase subunit C | 1.96 |
| bamD | PG1215 | outer membrane protein assembly factor BamD | 1.96 |
| PG_RS07980 | PG1810 | 2-oxoglutarate oxidoreductase | 1.94 |
| rpmD | PG1920 | 50S ribosomal protein L30 | 1.93 |
| PG_RS07075 | PG1605 | C1 family peptidase | 1.91 |
| PG_RS09300 | PG2097 | ribose-phosphate pyrophosphokinase | 1.90 |
| rnc | PG1763 | ribonuclease III | 1.85 |
| PG_RS07885 | PG1788 | aminopeptidase | 1.84 |
| PG_RS03915 | PG0886 | 2-amino-4-hydroxy-6-hydroxymethyldihydropteridine pyrophosphokinase | 1.82 |
| PG_RS05820 | PG1323 | PhoH family protein | 1.81 |
| PG_RS09335 | PG2106 | PorT family protein | 1.79 |
| PG_RS04695 | PG1067 | hypothetical protein | 1.73 |
| PG_RS01235 | PG0276 | Ceramide synthase (cerS) | 1.73 |
| nrfA | PG1820 | ammonia-forming cytochrome c nitrite reductase | 1.71 |
| PG_RS08815 | PG1998 | polyprenyl synthetase family protein | 1.68 |
| PG_RS05630 | PG1277 | nucleotide sugar dehydrogenase | 1.68 |
| PG_RS09330 | PG2105 | hypothetical protein | 1.67 |
| PG_RS05540 | PG1258 | integration host factor subunit beta | 1.66 |
| lon | PG0620 | ATP dependent protease La, endopeptidase | 1.64 |
| PG_RS05060 | PG1139 | DUF4369 domain-containing protein | 1.64 |
| dnaA | PG0001 | chromosomal replication initiator protein DnaA | 1.63 |
| trpS | PG2085 | tryptophan--tRNA ligase | 1.62 |
| PG_RS08235 | PG1878 | cysteine--tRNA ligase | 1.61 |
| PG_RS03110 | PG0708 | FKBP-type peptidyl-prolyl cis-trans isomerase | 1.61 |
| pckA | PG1676 | phosphoenolpyruvate carboxykinase (ATP) | 1.60 |
| PG_RS06145 | PG1396 | rod shape-determining protein | 1.60 |
| PG_RS09135 | PG2066 | DUF4837 family protein | 1.58 |
| PG_RS07420 | PG1683 | alpha-amylase | 1.58 |
| rnr | PG1721 | ribonuclease R | 1.57 |
| nrfH | PG1821 | cytochrome c nitrite reductase small subunit | 1.56 |
| rpmJ | PG1915 | 50S ribosomal protein L36 | 1.55 |
| PG_RS05795 | PG1316 | hypothetical protein | 1.54 |
| PG_RS07975 | PG1809 | 2-oxoglutarate ferredoxin oxidoreductase subunit gamma | 1.54 |
| PG_RS07095 | PG1610 | hypothetical protein | 1.54 |
| PG_RS05360 | PG1217 | DNA-directed RNA polymerase subunit omega | 1.53 |
| rplQ | PG1910 | 50S ribosomal protein L17 | 1.50 |
| PG_RS07435 |  | hypothetical protein | 1.44 |
| PG_RS07985 | PG1812 | 3-methyl-2-oxobutanoate dehydrogenase subunit VorB | 1.43 |
| PG_RS09250 | PG2086 | DUF3127 domain-containing protein | 1.37 |
| PG_RS07905 | PG1792 | sodium:hydrogen antiporter | 1.37 |
| PG_RS09720 | PG2189 | aspartate kinase | 1.34 |
| PG_RS09420 | PG2126 | YggS family pyridoxal phosphate-dependent enzyme | 1.32 |
| PG_RS09605 | PG2167 | DUF3868 domain-containing protein | 1.31 |
| PG_RS08415 | PG1911 | DNA-directed RNA polymerase subunit alpha | 1.31 |
| PG_RS04780 | PG1084 | AhpC/TSA family protein | 1.31 |
| gldE | PG0272 | gliding motility-associated protein GldE | 1.29 |
| PG_RS07415 | PG1682 | glycosyltransferase | 1.27 |
| PG_RS06965 | PG1583 | VWA domain-containing protein | 1.27 |
| PG_RS05955 | PG1353 | orotate phosphoribosyltransferase | 1.25 |
| PG_RS00445 | PG0099 | phenylalanine--tRNA ligase subunit beta | 1.25 |
| fabG | PG1239 | 3-oxoacyl-[acyl-carrier-protein] reductase | 1.23 |
| PG_RS09490 | PG2141 | ketoacyl-ACP synthase III | 1.23 |
| PG_RS07410 | PG1681 | 4-alpha-glucanotransferase | 1.22 |
| PG_RS04235 | PG0959 | Mrp/NBP35 family ATP-binding protein | 1.21 |
| omp28 | PG2173 | outer membrane lipoprotein Omp28 | 1.21 |
| PG_RS07035 | PG1597 | molecular chaperone DnaK | 1.21 |
| rpsN | PG1925 | 30S ribosomal protein S14 | 1.20 |
| rpsK | PG1913 | 30S ribosomal protein S11 | 1.19 |
| PG_RS09045 | PG2048 | hypothetical protein | 1.19 |
| PG_RS08090 | PG1837 | DUF2436 domain-containing protein | 1.19 |
| PG_RS01135 | PG0249 | oxaloacetate decarboxylase | 1.18 |
| PG_RS05970 | PG1356 | hypothetical protein | 1.17 |
| PG_RS05800 | PG1317 | hypothetical protein | 1.16 |
| pncB | PG0057 | nicotinate phosphoribosyltransferase | 1.14 |
| rpsD | PG1912 | 30S ribosomal protein S4 | 1.13 |
| nifJ | PG0548 | pyruvate:ferredoxin (flavodoxin) oxidoreductase | 1.13 |
| PG_RS03035 | PG0690 | acetyl-CoA hydrolase/transferase family protein | 1.12 |
| PG_RS07120 | PG1615 | fumarate reductase/succinate dehydrogenase flavoprotein | 1.11 |
| PG_RS04905 | PG1108 | Tellurite resistance protein TerB | 1.10 |
| pta | PG1082 | Phosphate acetyltransferase | 1.09 |
| rplC | PG1938 | 50S ribosomal protein L3 | 1.07 |
| PG_RS07660 | PG1741 | aspartate ammonia-lyase | 1.05 |
| meaB | PG0321 | methylmalonyl Co-A mutase-associated GTPase MeaB | 1.03 |
| rpsA | PG1297 | 30S ribosomal protein S1 | 1.00 |
| dnaJ | PG1776 | molecular chaperone DnaJ | -1.01 |
| PG_RS02090 | PG0471 | hypothetical protein | -1.01 |
| folE | PG0625 | GTP cyclohydrolase I FolE | -1.02 |
| porV | PG0027 | type IX secretion system outer membrane channel protein PorV | -1.02 |
| PG_RS01615 | PG0363 | transporter substrate-binding domain-containing protein | -1.03 |
| PG_RS02000 | PG0449 | tetratricopeptide repeat protein | -1.03 |
| PG_RS02635 | PG0594 | RNA polymerase sigma factor RpoD/SigA | -1.04 |
| PG_RS04485 | PG1017 | pyruvate 2C phosphate dikinase | -1.05 |
| rpmA | PG0315 | 50S ribosomal protein L27 | -1.05 |
| PG_RS02280 | PG0515 | alkaline phosphatase family protein | -1.06 |
| rgpB | PG0506 | Arg-gingipain RgpB | -1.07 |
| PG_RS01610 | PG0362 | putative porin | -1.07 |
| sufC | PG0258 | Fe-S cluster assembly ATPase SufC | -1.08 |
| PG_RS01285 | PG0287 | type IX secretion system membrane protein PorP/SprF | -1.14 |
| PG_RS02960 | PG0674 | indolepyruvate oxidoreductase subunit beta | -1.18 |
| carA | PG0529 | glutamine-hydrolyzing carbamoyl-phosphate synthase | -1.21 |
| PG_RS02480 | PG0558 | purine nucleoside phosphorylase | -1.22 |
| PG_RS06820 | PG1545 | superoxide dismutase | -1.22 |
| PG_RS02630 | PG0593 | DegQ family serine endoprotease | -1.22 |
| PG_RS00290 | PG0063 | TolC family protein | -1.22 |
| PG_RS08055 | PG1827 | RNA polymerase sigma factor | -1.25 |
| PG_RS07270 | PG1653 | HAD-IIB family hydrolase | -1.25 |
| PG_RS02805 | PG0634 | DJ-1/PfpI family protein | -1.26 |
| PG_RS07275 | PG1654 | M15 family metallopeptidase | -1.26 |
| PG_RS05670 | PG1286 | ferritin | -1.26 |
| PG_RS09845 | PG2215 | mannose-1-phosphate guanylyltransferase | -1.30 |
| PG_RS05815 | PG1321 | formate--tetrahydrofolate ligase | -1.30 |
| PG_RS00790 | PG0172 | 3'-5' exonuclease | -1.30 |
| PG_RS07820 | PG1777 | DUF59 domain-containing protein | -1.31 |
| pepT | PG0445 | peptidase T | -1.33 |
| PG_RS00405 | PG0090 | DNA starvation/stationary phase protection protein | -1.34 |
| PG_RS09730 | PG2192 | peptidoglycan DD-metalloendopeptidase family protein | -1.35 |
| PG_RS03335 | PG0759 | tetratricopeptide repeat protein | -1.36 |
| PG_RS01210 | PG0270 | hydrogen peroxide-inducible genes activator | -1.37 |
| gcvP | PG1305 | aminomethyl-transferring glycine dehydrogenase | -1.39 |
| PG_RS09765 | PG2200 | tetratricopeptide repeat protein | -1.40 |
| obgE | PG0790 | GTPase ObgE | -1.41 |
| PG_RS01290 | PG0288 | SUMF1/EgtB/PvdO family nonheme iron enzyme | -1.43 |
| PG_RS08170 | PG1858 | flavodoxin | -1.44 |
| PG_RS05875 | PG1334 | SPFH/Band 7/PHB domain protein | -1.46 |
| sufD | PG0259 | Fe-S cluster assembly protein SufD | -1.47 |
| ruvA | PG0811 | Holliday junction branch migration protein RuvA | -1.47 |
| gldL | PG0289 | gliding motility protein GldL | -1.48 |
| PG_RS08575 | PG1944 | 3-phosphoshikimate 1-carboxyvinyltransferase | -1.48 |
| PG_RS08585 | PG1946 | metal ABC transporter permease | -1.49 |
| PG_RS09785 | PG2205 | 2-dehydropantoate 2-reductase | -1.51 |
| PG_RS04080 | PG0928 | PglZ domain-containing protein | -1.53 |
| PG_RS00375 | PG0084 | L-serine ammonia-lyase | -1.54 |
| PG_RS00215 | PG0046 | phosphatidate cytidylyltransferase | -1.55 |
| pruA | PG1269 | L-glutamate gamma-semialdehyde dehydrogenase | -1.55 |
| PG_RS07325 | PG1666 | efflux RND transporter periplasmic adaptor subunit | -1.57 |
| PG_RS01450 | PG0323 | cupin domain-containing protein | -1.59 |
| dnaK | PG1208 | molecular chaperone DnaK | -1.62 |
| xth | PG0269 | exodeoxyribonuclease III | -1.64 |
| PG_RS07310 | PG1663 | ABC transporter ATP-binding protein | -1.66 |
| PG_RS01180 | PG0263 | tyrosine--tRNA ligase | -1.68 |
| PG_RS00055 | PG0010 | ATP-dependent Clp protease ATP-binding subunit | -1.69 |
| PG_RS02310 | PG0521 | co-chaperone GroES | -1.70 |
| PG_RS01185 | PG0264 | glycosyltransferase family 2 protein | -1.78 |
| PG_RS00360 | PG0081 | hypothetical protein | -1.79 |
| gldM | PG0290 | gliding motility protein GldM | -1.84 |
| PG_RS02800 | PG0633 | TonB family protein | -1.85 |
| groL | PG0520 | chaperonin GroEL | -1.85 |
| tsaE | PG0927 | tRNA (adenosine(37)-N6)-threonylcarbamoyltransferase complex ATPase subunit type 1 TsaE | -1.86 |
| PG_RS08255 | PG1884 | alpha-L-fucosidase | -1.87 |
| PG_RS01865 | PG0419 | DUF2807 domain-containing protein | -1.87 |
| PG_RS07230 |  | heavy-metal-associated domain-containing protein | -1.91 |
| ahpC | PG0618 | peroxiredoxin | -1.94 |
| PG_RS01035 | PG0226 | transglutaminase domain-containing protein | -1.98 |
| PG_RS07265 | PG1652 | hypothetical protein | -1.99 |
| PG_RS02385 | PG0534 | TonB-dependent receptor | -2.02 |
| PG_RS04800 | PG1089 | response regulator transcription factor | -2.02 |
| PG_RS03295 | PG0751 | PorT family protein | -2.03 |
| gldN | PG0291 | gliding motility protein GldN | -2.05 |
| PG_RS00110 | PG0024 | redox-sensing transcriptional repressor Rex | -2.15 |
| PG_RS01490 | PG0332 | transcription termination factor Rho | -2.19 |
| PG_RS02055 | PG0462 | MFS transporter | -2.20 |
| PG_RS07225 | PG1642 | copper-translocating P-type ATPase | -2.21 |
| PG_RS07305 | PG1662 | hypothetical protein | -2.22 |
| PG_RS04660 | PG1058 | OmpA family protein | -2.27 |
| deoC | PG1996 | deoxyribose-phosphate aldolase | -2.28 |
| PG_RS07930 | PG1798 | T9SS type A sorting domain-containing protein | -2.33 |
| PG_RS08580 | PG1945 | hypothetical protein | -2.34 |
| PG_RS01475 | PG0328 | imidazolonepropionase | -2.40 |
| PG_RS03290 | PG0750 | glycosyltransferase | -2.40 |
| PG_RS02260 | PG0511 | membrane protein | -2.40 |
| PG_RS08590 | PG1947 | tetratricopeptide repeat protein | -2.41 |
| queA | PG1540 | tRNA preQ1(34) S-adenosylmethionine ribosyltransferase-isomerase QueA | -2.46 |
| PG_RS08595 | PG1948 | alpha/beta hydrolase | -2.51 |
| secA | PG0514 | preprotein translocase subunit SecA | -2.55 |
| PG_RS06595 | PG1497 | hypothetical protein | -2.61 |
| PG_RS07330 | PG1667 | TolC family protein | -2.62 |
| hflB | PG0047 | ATP-dependent metallopeptidase FtsH/Yme1/Tma family protein | -2.69 |
| PG_RS00115 | PG0025 | fumarylacetoacetate hydrolase family protein | -2.71 |
| PG_RS03865 | PG0875 | helix-turn-helix domain-containing protein | -2.73 |
| PG_RS01310 |  | chromate transporter | -2.76 |
| PG_RS02285 | PG0516 | DUF4105 domain-containing protein | -2.92 |
| htpG | PG0045 | molecular chaperone HtpG | -3.00 |
| porU | PG0026 | type IX secretion system sortase PorU | -3.02 |
| ahpF | PG0619 | alkyl hydroperoxide reductase subunit F | -3.03 |
| PG_RS07950 | PG1804 | V-type ATP synthase subunit B | -3.06 |
| PG_RS02595 | PG0585 | GatB/YqeY domain-containing protein; tRNA metabolism | -3.23 |
| PG_RS02420 | PG0541 | hypothetical protein | -3.45 |
| PG_RS03150 | PG0718 | hypothetical protein | -3.47 |
| PG_RS01080 | PG0236 | hypothetical protein | -3.69 |
| PG_RS07170 | PG1625 | hypothetical protein | -3.76 |
| radA | PG0227 | DNA repair protein RadA | -3.95 |
| PG_RS06665 | PG1512 | DNA helicase | -3.96 |
| PG_RS01870 | PG0421 | DUF2807 domain-containing protein | -4.00 |
| PG_RS03835 | PG0870 | hypothetical protein | -4.14 |
| PG_RS06585 | PG1495 | type IA DNA topoisomerase | -4.49 |
| PG_RS03840 | PG0871 | DUF3987 domain-containing protein | -4.50 |
| PG_RS02415 | PG0540 | efflux RND transporter permease subunit | -4.67 |
| PG_RS07175 | PG1626 | transporter | -4.79 |
| PG_RS03020 | PG0686 | DUF1858 domain-containing protein | -5.52 |
| PG_RS03145 | PG0717 | hypothetical protein | -5.63 |
