# Supplementary material for: Growth of Porphyromonas gingivalis on human serum albumin triggers programmed cell death
Source: J Oral Microbiol. 2022 Dec 22;15(1):2161182. doi: 10.1080/20002297.2022.2161182 (PMC9788703; doi:10.1080/20002297.2022.2161182)
Supplement: Supplemental Material [file ZJOM_A_2161182_SM7719.zip › supplementary files/Supplemental Table S1c.docx]

**Supplemental Table S1c.** Differential gene expression analyzed by pairwise comparison of the transcriptomes of strain **W83 at 15.5 hrs** with **W83 at 12.5hrs**. Gene number and predicted function are provided. Highlighted in green are genes that are discussed in the manuscript. (Fold change ≥ 2; *q*-value < 0.01)

| Name | oldName | Annotation | **logFC** |
| --- | --- | --- | --- |
| PG_RS00900 | PG0195 | rubrerythrin family protein | -9.98 |
| PG_RS02400 | PG0537 | aminoacyl-histidine dipeptidase | -6.04 |
| PG_RS10480 | PG1421 | 4Fe-4S binding protein | -5.54 |
| PG_RS07310 | PG1663 | ABC transporter ATP-binding protein | -5.37 |
| PG_RS07255 | PG1648 | bifunctional (p)ppGpp synthetase/ hydrolase | -4.98 |
| PG_RS07325 | PG1666 | efflux RND transporter periplasmic adaptor subunit | -4.78 |
| PG_RS05500 | PG1249 | 1-acyl-sn-glycerol-3-phosphate acyltransferase | -4.70 |
| PG_RS05980 | PG1358 | GNAT family N-acetyltransferase | -4.48 |
| PG_RS02655 | PG0598 | YjgP/YjgQ family permease | -4.48 |
| PG_RS06465 | PG1471 | PH domain-containing protein | -4.48 |
| PG_RS00290 | PG0063 | TolC family protein | -4.45 |
| scpA | PG1657 | methylmalonyl-CoA mutase | -4.39 |
| pssA | PG0964 | CDP-diacylglycerol--serine O-phosphatidyltransferase | -4.35 |
| PG_RS02710 | PG0614 | hypothetical protein | -4.35 |
| PG_RS05895 | PG1340 | L-lactate permease | -4.20 |
| PG_RS04995 | PG1127 | winged helix-turn-helix transcriptional regulator | -4.20 |
| PG_RS01820 | PG0410 | T9SS type A sorting domain-containing protein | -4.06 |
| PG_RS03665 | PG0834 | DUF4297 domain-containing protein | -4.04 |
| PG_RS02540 | PG0574 | hypothetical protein | -4.04 |
| PG_RS04935 | PG1114 | aspartate 1-decarboxylase | -4.04 |
| PG_RS03150 | PG0718 | hypothetical protein | -4.04 |
| PG_RS10650 |  | hypothetical protein | -3.96 |
| PG_RS01115 | PG0245 | universal stress protein | -3.91 |
| PG_RS05805 | PG1318 | sigma-70 family RNA polymerase sigma factor | -3.88 |
| PG_RS06025 | PG1367 | DUF4290 domain-containing protein | -3.86 |
| PG_RS07125 | PG1616 | succinate dehydrogenase/fumarate reductase | -3.22 |
| PG_RS09780 | PG2204 | alpha-2-macroglobulin family protein | -3.22 |
| PG_RS02700 | PG0611 | hypothetical protein | -3.16 |
| PG_RS07115 | PG1614 | succinate dehydrogenase/fumarate reductase iron-sulfur | -3.13 |
| PG_RS00295 | PG0064 | CusA/CzcA family heavy metal efflux RND transporter | -3.08 |
| aroC | PG1314 | chorismate synthase | -3.06 |
| PG_RS07975 | PG1809 | 2-oxoglutarate ferredoxin oxidoreductase subunit gamma | -3.00 |
| PG_RS05630 | PG1277 | nucleotide sugar dehydrogenase | -2.99 |
| PG_RS03145 | PG0717 | hypothetical protein | -2.92 |
| PG_RS07980 | PG1810 | 2-oxoglutarate oxidoreductase | -2.92 |
| PG_RS06575 | PG1493 | hypothetical protein | -2.86 |
| bamD | PG1215 | outer membrane protein assembly factor BamD | -2.84 |
| PG_RS07420 | PG1683 | alpha-amylase | -2.83 |
| PG_RS07120 | PG1615 | fumarate reductase/succinate dehydrogenase flavoprotein | -2.79 |
| PG_RS01025 | PG0224 | MarC family protein | -2.79 |
| PG_RS07710 | PG1754 | S9 family peptidase | -2.78 |
| PG_RS02770 | PG0627 | RNA-binding protein | -2.73 |
| PG_RS07905 | PG1792 | sodium:hydrogen antiporter | -2.72 |
| PG_RS05945 | PG1351 | hypothetical protein | -2.65 |
| PG_RS09045 | PG2048 | hypothetical protein | -2.65 |
| PG_RS07990 | PG1813 | 4Fe-4S dicluster domain-containing protein | -2.62 |
| trkA | PG2218 | Trk system potassium transporter TrkA | -2.56 |
| PG_RS03440 | PG0784 | polyprenyl synthetase family protein | -2.55 |
| PG_RS04535 | PG1030 | T9SS type A sorting domain-containing protein | -2.52 |
| nrfA | PG1820 | ammonia-forming cytochrome c nitrite reductase | -2.50 |
| PG_RS07480 | PG1697 | DEAD/DEAH box helicase family protein | -2.46 |
| PG_RS08090 | PG1837 | DUF2436 domain-containing protein | -2.45 |
| PG_RS02080 | PG0468 | mannose-6-phosphate isomerase | -2.44 |
| PG_RS05065 | PG1140 | glycosyltransferase family 2 protein | -2.36 |
| PG_RS06220 | PG1414 | TonB-dependent receptor plug domain-containing protein | -2.35 |
| PG_RS07985 | PG1812 | 3-methyl-2-oxobutanoate dehydrogenase subunit VorB | -2.35 |
| PG_RS09840 | PG2214 | DUF2851 family protein | -2.29 |
| PG_RS07475 | PG1696 | hypothetical protein | -2.29 |
| PG_RS01235 | PG0276 | Ceramide synthase (*cerS*) | -2.28 |
| PG_RS01390 | PG0310 | nitroreductase | -2.28 |
| ssb | PG0271 | single-stranded DNA-binding protein | -2.28 |
| mscL | PG1330 | large-conductance mechanosensitive channel protein MscL | -2.18 |
| PG_RS05820 | PG1323 | PhoH family protein | -2.18 |
| PG_RS09720 | PG2189 | aspartate kinase | -2.11 |
| PG_RS01320 | PG0293 | N-acetylmuramidase | -2.11 |
| nrfH | PG1821 | cytochrome c nitrite reductase small subunit | -2.10 |
| ychF | PG0048 | redox-regulated ATPase YchF | -2.09 |
| PG_RS06965 | PG1583 | VWA domain-containing protein | -2.08 |
| PG_RS09725 | PG2190 | ATP-binding cassette domain-containing protein | -2.06 |
| PG_RS06565 | PG1491 | hypothetical protein | -2.04 |
| PG_RS09555 | PG2155 | TraB/GumN family protein | -2.03 |
| PG_RS07910 | PG1793 | 1 2C4-alpha-glucan-branching protein | -2.01 |
| PG_RS08970 | PG2031 | hypothetical protein | -2.01 |
| PG_RS05775 | PG1313 | peptidase C69 | -2.00 |
| PG_RS07920 | PG1795 | hypothetical protein | -1.90 |
| PG_RS08610 | PG1950 | AI-2E family transporter | -1.84 |
| PG_RS07260 | PG1651 | tetratricopeptide repeat protein | -1.81 |
| PG_RS05330 | PG1210 | aminopeptidase P family protein | -1.80 |
| PG_RS03435 | PG0783 | TatD family hydrolase | -1.77 |
| PG_RS05955 | PG1353 | orotate phosphoribosyltransferase | -1.76 |
| PG_RS09755 | PG2198 | hypothetical protein | -1.75 |
| PG_RS05875 | PG1334 | SPFH/Band 7/PHB domain protein | -1.71 |
| PG_RS06455 | PG1469 | N-6 DNA methylase | -1.69 |
| PG_RS01825 | PG0411 | T9SS type A sorting domain-containing protein | -1.68 |
| PG_RS05610 | PG1270 | amidinotransferase | -1.67 |
| PG_RS08340 | PG1896 | methionine adenosyltransferase | -1.65 |
| PG_RS01195 | PG0267 | arginine--tRNA ligase | -1.64 |
| PG_RS05060 | PG1139 | DUF4369 domain-containing protein | -1.63 |
| PG_RS01315 |  | chromate transporter | -1.63 |
| PG_RS06960 | PG1582 | VWA domain-containing protein | -1.61 |
| PG_RS06230 | PG1417 | fumarate hydratase | -1.59 |
| gldE | PG0272 | gliding motility-associated protein GldE | -1.54 |
| PG_RS07410 | PG1681 | 4-alpha-glucanotransferase | -1.51 |
| pruA | PG1269 | L-glutamate gamma-semialdehyde dehydrogenase | -1.51 |
| PG_RS04650 | PG1056 | 6-carboxytetrahydropterin synthase | -1.51 |
| PG_RS03100 | PG0706 | META domain-containing protein | -1.47 |
| PG_RS07150 | PG1622 | DNA gyrase/topoisomerase IV subunit A | -1.44 |
| PG_RS05650 | PG1281 | DUF2027 domain-containing protein | -1.43 |
| PG_RS07425 | PG1684 | hypothetical protein | -1.38 |
| PG_RS02980 | PG0678 | isochorismatase family protein | -1.38 |
| murA | PG1366 | UDP-N-acetylglucosamine 1-carboxyvinyltransferase | -1.38 |
| PG_RS04105 | PG0933 | elongation factor G | -1.36 |
| PG_RS07415 | PG1682 | glycosyltransferase | -1.36 |
| PG_RS01135 | PG0249 | oxaloacetate decarboxylase | -1.35 |
| PG_RS03400 | PG0777 | electron transfer flavoprotein subunit beta/FixA family protein | -1.34 |
| PG_RS05795 | PG1316 | hypothetical protein | -1.33 |
| PG_RS04905 | PG1108 | Tellurite resistance protein TerB | -1.31 |
| PG_RS05260 | PG1189 | DUF349 domain-containing protein | -1.31 |
| frr | PG1901 | ribosome recycling factor | -1.29 |
| fabD | PG0138 | ACP S-malonyltransferase | -1.29 |
| PG_RS09335 | PG2106 | PorT family protein | -1.27 |
| PG_RS06975 | PG1585 | protein BatD | -1.25 |
| purD | PG1360 | phosphoribosylamine--glycine ligase | -1.24 |
| PG_RS02280 | PG0515 | alkaline phosphatase family protein | -1.22 |
| PG_RS05115 | PG1151 | iron-containing alcohol dehydrogenase | -1.22 |
| murB | PG1342 | UDP-N-acetylmuramate dehydrogenase | -1.20 |
| PG_RS05950 | PG1352 | hypothetical protein | -1.19 |
| PG_RS07550 | PG1715 | TonB-dependent receptor | -1.19 |
| PG_RS07270 | PG1653 | HAD-IIB family hydrolase | -1.18 |
| PG_RS01800 | PG0404 | hypothetical protein | -1.17 |
| PG_RS07715 | PG1755 | fructose bisphosphate aldolase | -1.17 |
| PG_RS00425 | PG0094 | TolC family protein | -1.14 |
| PG_RS07485 | PG1701 | gamma-glutamyl-gamma-aminobutyrate hydrolase family protein | -1.14 |
| PG_RS07435 |  | hypothetical protein | -1.12 |
| PG_RS00635 | PG0137 | aminoacyl-histidine dipeptidase | -1.11 |
| dxs | PG2217 | 1-deoxy-D-xylulose-5-phosphate synthase | -1.11 |
| galK | PG1633 | galactokinase | -1.07 |
| PG_RS09105 | PG2060 | thymidylate synthase | -1.04 |
| PG_RS06570 | PG1492 | GLPGLI family protein | -1.02 |
| PG_RS08600 | PG1949 | malate dehydrogenase | -1.01 |
| PG_RS00750 | PG0162 | sigma-70 family RNA polymerase sigma factor | -1.01 |
| PG_RS00445 | PG0099 | phenylalanine--tRNA ligase subunit beta | -1.01 |
| rpsR | PG0596 | 30S ribosomal protein S18 | 1.00 |
| PG_RS04745 | PG1077 | electron transfer flavoprotein subunit beta/FixA family protein | 1.01 |
| PG_RS08595 | PG1948 | alpha/beta hydrolase | 1.02 |
| lon | PG0620 | ATP dependent protease La, endopeptidase | 1.02 |
| PG_RS04740 | PG1076 | acyl-CoA dehydrogenase | 1.04 |
| sufB | PG0257 | Fe-S cluster assembly protein SufB | 1.10 |
| nusG | PG0389 | transcription termination/antitermination factor NusG | 1.10 |
| PG_RS03925 | PG0890 | alkaline phosphatase | 1.10 |
| PG_RS05815 | PG1321 | formate--tetrahydrofolate ligase | 1.13 |
| PG_RS02335 | PG0528 | amidophosphoribosyltransferase | 1.14 |
| PG_RS06840 | PG1551 | heme-binding protein HmuY | 1.15 |
| secE |  | preprotein translocase subunit SecE | 1.17 |
| PG_RS03470 | PG0791 | adenylate kinase | 1.18 |
| PG_RS02185 | PG0491 | S46 family peptidase | 1.19 |
| rplL | PG0393 | 50S ribosomal protein L7/L12 | 1.19 |
| PG_RS00310 | PG0069 | bifunctional ADP-dependent NAD(P)H-hydrate dehydratase/NAD(P)H-hydrate epimerase | 1.22 |
| PG_RS03110 | PG0708 | FKBP-type peptidyl-prolyl cis-trans isomerase | 1.28 |
| PG_RS04475 | PG1013 | acetyl-CoA hydrolase/transferase family protein | 1.29 |
| PG_RS08255 | PG1884 | alpha-L-fucosidase | 1.30 |
| recN | PG1849 | DNA repair protein RecN | 1.33 |
| PG_RS02650 | PG0597 | 50S ribosomal protein L9 | 1.39 |
| PG_RS06845 | PG1552 | TonB-dependent receptor | 1.39 |
| PG_RS02640 | PG0595 | 30S ribosomal protein S6 | 1.41 |
| PG_RS07935 | PG1801 | hypothetical protein | 1.45 |
| PG_RS04485 | PG1017 | pyruvate 2C phosphate dikinase | 1.46 |
| rpmJ | PG1915 | 50S ribosomal protein L36 | 1.47 |
| carA | PG0529 | glutamine-hydrolyzing carbamoyl-phosphate synthase | 1.48 |
| PG_RS02530 | PG0571 | aspartate-semialdehyde dehydrogenase | 1.49 |
| PG_RS08155 | PG1855 | S41 family peptidase | 1.51 |
| PG_RS02460 | PG0555 | histidinol phosphate phosphatase | 1.52 |
| PG_RS01740 | PG0391 | 50S ribosomal protein L1 | 1.53 |
| PG_RS01745 | PG0392 | 50S ribosomal protein L10 | 1.56 |
| ahpC | PG0618 | peroxiredoxin | 1.61 |
| PG_RS00055 | PG0010 | ATP-dependent Clp protease ATP-binding subunit | 1.62 |
| rpsA | PG1297 | 30S ribosomal protein S1 | 1.63 |
| PG_RS03965 | PG0900 | cytochrome ubiquinol oxidase subunit I | 1.65 |
| sufD | PG0259 | Fe-S cluster assembly protein SufD | 1.65 |
| dnaJ | PG1776 | molecular chaperone DnaJ | 1.66 |
| rpsG | PG1941 | 30S ribosomal protein S7 | 1.70 |
| rplQ | PG1910 | 50S ribosomal protein L17 | 1.71 |
| rpmD | PG1920 | 50S ribosomal protein L30 | 1.74 |
| rpsK | PG1913 | 30S ribosomal protein S11 | 1.74 |
| PG_RS07230 |  | heavy-metal-associated domain-containing protein | 1.74 |
| PG_RS03115 | PG0709 | FKBP-type peptidyl-prolyl cis-trans isomerase | 1.75 |
| rpsH | PG1924 | 30S ribosomal protein S8 | 1.75 |
| rpmH | PG0656 | 50S ribosomal protein L34 | 1.75 |
| rplK | PG0390 | 50S ribosomal protein L11 | 1.79 |
| PG_RS01230 | PG0275 | redoxin domain-containing protein | 1.84 |
| map | PG1917 | type I methionyl aminopeptidase | 1.86 |
| rpsE | PG1921 | 30S ribosomal protein S5 | 1.88 |
| secY | PG1918 | preprotein translocase subunit SecY | 1.91 |
| PG_RS02055 | PG0462 | MFS transporter | 1.92 |
| nqrF | PG2177 | NADH:ubiquinone reductase (Na(+)-transporting) subunit F | 1.93 |
| rpoC | PG0395 | DNA-directed RNA polymerase subunit beta' | 1.94 |
| PG_RS07820 | PG1777 | DUF59 domain-containing protein | 1.96 |
| rpsM | PG1914 | 30S ribosomal protein S13 | 1.97 |
| sufC | PG0258 | Fe-S cluster assembly ATPase SufC | 1.97 |
| PG_RS01460 | PG0325 | cyclodeaminase/cyclohydrolase family protein | 1.98 |
| rplF | PG1923 | 50S ribosomal protein L6 | 1.99 |
| ptk1 | PG0436 | polysaccharide biosynthesis tyrosine autokinase Ptk1 | 2.01 |
| PG_RS08465 | PG1922 | 50S ribosomal protein L18 | 2.03 |
| rpsD | PG1912 | 30S ribosomal protein S4 | 2.06 |
| PG_RS08625 | PG1953 | YitT family protein | 2.08 |
| PG_RS01930 | PG0434 | hypothetical protein | 2.09 |
| rplO | PG1919 | 50S ribosomal protein L15 | 2.09 |
| ftcD | PG0329 | glutamate formimidoyltransferase | 2.09 |
| PG_RS00775 | PG0167 | 50S ribosomal protein L25/general stress protein Ctc | 2.11 |
| PG_RS08415 | PG1911 | DNA-directed RNA polymerase subunit alpha | 2.16 |
| rgpB | PG0506 | Arg-gingipain RgpB | 2.17 |
| PG_RS07225 | PG1642 | copper-translocating P-type ATPase | 2.19 |
| hutH | PG0324 | histidine ammonia-lyase | 2.22 |
| PG_RS07170 | PG1625 | hypothetical protein | 2.23 |
| rpoB | PG0394 | DNA-directed RNA polymerase subunit beta | 2.25 |
| PG_RS07945 | PG1803 | V-type ATP synthase subunit A | 2.29 |
| rplM | PG0375 | 50S ribosomal protein L13 | 2.29 |
| PG_RS07810 | PG1775 | nucleotide exchange factor GrpE | 2.35 |
| PG_RS04630 | PG1051 | O-antigen ligase family protein | 2.39 |
| PG_RS03105 | PG0707 | TonB-dependent receptor | 2.40 |
| PG_RS07175 | PG1626 | transporter | 2.43 |
| PG_RS08565 | PG1942 | 30S ribosomal protein S12 | 2.50 |
| PG_RS06850 | PG1553 | cobaltochelatase subunit CobN | 2.55 |
| dnaK | PG1208 | molecular chaperone DnaK | 2.58 |
| hflB | PG0047 | ATP-dependent metallopeptidase FtsH/Yme1/Tma family protein | 2.58 |
| PG_RS02410 | PG0539 | efflux RND transporter periplasmic adaptor subunit | 2.60 |
| PG_RS07960 | PG1806 | ATPase | 2.61 |
| rpsN | PG1925 | 30S ribosomal protein S14 | 2.61 |
| groL | PG0520 | chaperonin GroEL | 2.63 |
| infA | PG1916 | translation initiation factor IF-1 | 2.71 |
| rpsC | PG1932 | 30S ribosomal protein S3 | 2.75 |
| PG_RS02310 | PG0521 | co-chaperone GroES | 2.77 |
| rpsQ | PG1929 | 30S ribosomal protein S17 | 2.81 |
| PG_RS07955 | PG1805 | V-type ATP synthase subunit D | 2.81 |
| PG_RS07965 | PG1807 | ATP synthase subunit C | 2.85 |
| rplV | PG1933 | 50S ribosomal protein L22 | 2.91 |
| PG_RS06055 | PG1374 | T9SS type A sorting domain-containing protein | 2.92 |
| PG_RS08170 | PG1858 | flavodoxin | 2.99 |
| pheS | PG1771 | phenylalanine--tRNA ligase subunit alpha | 2.99 |
| rpsB | PG0377 | 30S ribosomal protein S2 | 3.04 |
| rpsI | PG0376 | 30S ribosomal protein S9 | 3.10 |
| PG_RS02405 | PG0538 | TolC family protein | 3.12 |
| rplC | PG1938 | 50S ribosomal protein L3 | 3.12 |
| PG_RS01475 | PG0328 | imidazolonepropionase | 3.18 |
| clpB | PG1118 | ATP-dependent chaperone ClpB | 3.19 |
| rpmC | PG1930 | 50S ribosomal protein L29 | 3.20 |
| rpsJ | PG1939 | 30S ribosomal protein S10 | 3.24 |
| PG_RS02195 | PG0495 | T9SS type A sorting domain-containing protein | 3.29 |
| rplB | PG1935 | 50S ribosomal protein L2 | 3.32 |
| rplE | PG1926 | 50S ribosomal protein L5 | 3.37 |
| rpsS | PG1934 | 30S ribosomal protein S19 | 3.46 |
| PG_RS01665 | PG0378 | elongation factor Ts | 3.52 |
| rplP | PG1931 | 50S ribosomal protein L16 | 3.54 |
| fusA | PG1940 | elongation factor G | 3.54 |
| PG_RS07950 | PG1804 | V-type ATP synthase subunit B | 3.55 |
| PG_RS08165 | PG1857 | DUF2023 family protein | 3.58 |
| rplX | PG1927 | 50S ribosomal protein L24 | 3.64 |
| PG_RS02420 | PG0541 | hypothetical protein | 3.67 |
| rplD | PG1937 | 50S ribosomal protein L4 | 3.71 |
| rplN | PG1928 | 50S ribosomal protein L14 | 3.78 |
| rplW | PG1936 | 50S ribosomal protein L23 | 3.80 |
| PG_RS03165 | PG0721 | C40 family peptidase | 3.89 |
| udk | PG1781 | uridine kinase | 3.90 |
| PG_RS03020 | PG0686 | DUF1858 domain-containing protein | 4.10 |
| ahpF | PG0619 | alkyl hydroperoxide reductase subunit F | 4.13 |
| PG_RS07940 | PG1802 | DUF2764 domain-containing protein | 4.28 |
| PG_RS01870 | PG0421 | DUF2807 domain-containing protein | 4.34 |
| PG_RS00515 | PG0111 | capsular polysaccharide biosynthesis protein | 4.36 |
| htpG | PG0045 | molecular chaperone HtpG | 4.50 |
| PG_RS02415 | PG0540 | efflux RND transporter permease subunit | 5.09 |
| PG_RS11080 |  | DUF1661 domain-containing protein | 5.42 |
