# Supplementary material for: Growth of Porphyromonas gingivalis on human serum albumin triggers programmed cell death
Source: J Oral Microbiol. 2022 Dec 22;15(1):2161182. doi: 10.1080/20002297.2022.2161182 (PMC9788703; doi:10.1080/20002297.2022.2161182)
Supplement: Supplemental Material [file ZJOM_A_2161182_SM7719.zip › supplementary files/Supplemental Table S2_antisense.docx]

**Supplemental Table S2**.  Differential expression of **antisense RNA** analyzed by transcriptomic comparison of strain W83 with W50 (Fold change ≥ 2; *q*-value < 0.01)

| **Differential gene expression: early lysis and late exponential phase in W83 strain**  **analyzed by** **Rockhopper** | | | | | | | |  |
| --- | --- | --- | --- | --- | --- | --- | --- | --- |
| **predicted asRNA target genes** | **Gene name** | **Locus region of gene** | | **Transcriptional location of predicted asRNA** | | **Predicted function/**  **pathway** | **Fold changes** |  |
|  |  | **Start** | **Stop** | **Start** | **Stop** |  |  |  |
| antisense: PG1551 | hmuY | 1628578 | 1629006 | 1629014 | 1628353 |  | 0.15 |  |
| antisense: PG1704 | thiol:disulfide interchange protein dsbD, Putative | 1790861 | 1792909 | 1790873 | 1790890 |  | 0.57 |  |
| antisense: PG1858 | flavodoxin | 1956628 | 1957125 | 1956676 | 1957121 | iron ion binding | 0.09 |  |
| antisense: PG0263 | tyrosyl-tRNA synthetase )(tyrS) | 295803 | 297095 | 296471 | 296460 |  | 0.61 |  |
| **Differential gene expression: early lysis and late exponential phase in W83**  **analyzed by Degust** | | | | | | | |  |
| **predicted asRNA target genes** | **Gene name** | **Locus region of gene** | | **Predicted function/pathway** | | | **Fold changes** |  |
|  |  | **Start** | **Stop** |  |  |  |  |  |
| antisense: PG1807 | v-type ATPase, subunit K (atpK) | 1902209 | 1902685 | Bioenergetics | | | 0.13 |  |
| antisense: PG0138 | malonyl CoA-acyl carrier protein transacylase (fabD) | 160813 | 161694 | fatty acid biosynthetic process | | | 0.13 |  |
| antisense: PG0669/ PG0668 | PG0669: heme-binding protein FetB/ PG0668: TonB-dependent receptor | 719893/ 717591 | 720774/ 719864 | PG0669: sirohydrochlorin cobaltochelatase activity/ PG0668: transporter activity | | | 0.13 |  |
| antisense: PG1559 | glycine cleavage system T protein (gcvT) | 1637994 | 1639082 | glycine catabolic process | | | 0.14 |  |
| antisense: PG1551 | hmuY | 1628578 | 1629006 |  | | | 0.15 |  |
| antisense: PG1609/ PG1608 | PG1609: methylmalonyl-CoA decarboxylase, gamma subunit (mmdC)/ PG1608 : methylmalonyl-CoA decarboxylase, beta subunit (mmdB) | 1689931/ 1688775 | 1690365/ 1689926 | PG1609: biotin binding/ PG1608: sodium ion transport, lyase activity, oxaloacetate decarboxylase activity | | | 0.15 |  |
| antisense: PG0137 | aminoacyl-histidine dipeptidase (pepD-1) | 159244 | 160698 |  | | | 0.15 |  |
| antisense: PG0137 |  |  |  |  | | | 0.17 |  |
| antisense: PG0323 | conserved hypothetical protein | 357149 | 357478 |  | | | 0.17 |  |
| antisense: PG0933 | translation elongation factor G, putative | 990137 | 992296 | small GTP-binding protein domain, protein-synthesizing GTPase activity | | | 0.17 |  |
| antisense: PG0933 |  |  |  |  |  |  | 0.177 |  |
| antisense: PG1189 | hypothetical protein | 1270132 | 1272012 |  | | | 0.18 |  |
| antisense: PG1704 | thiol:disulfide interchange protein dsbD, Putative | 1790861 | 1792909 |  | | | 0.18 |  |
| antisense: PG1704 |  |  |  |  | | | 0.18 |  |
| antisense: PG1704 |  |  |  |  | | | 0.19 |  |
| antisense: PG1858 | flavodoxin | 1956628 | 1957125 |  | | | 0.19 |  |
| antisense: PG2082 | Oligopeptide transporter | 2181933 | 2183474 | Nutrient acquisition | | | 0.19 |  |
| antisense: PG2082 |  |  |  |  |  |  | 0.19 |  |
| antisense: PG0506 | arginine-specific cysteine proteinase (RgpB) | 545475 | 547685 | Protease gingipain-Nutrient acquisition | | | 0.19 |  |
| antisense: PG1911 | DNA-directed RNA polymerase, alpha subunit (rpoA) | 2010860 | 2011852 | transcription, DNA-dependent | | | 0.2 |  |
| antisense: PG1914 | ribosomal protein S13 (rpsM) | 2013028 | 2013408 | RNA binding | | | 0.2 |  |
| antisense: PG1762 | bifunctional preprotein translocase subunit SecD/SecF (secDF) | 1848017 | 1850962 | Membrane protein secretion system | | | 0.2 |  |
| antisense: PG1762 |  |  |  |  |  |  | 0.2 |  |
| antisense: PG1762 |  |  |  |  |  |  | 0.2 |  |
| antisense: PG1401 | beta-eliminating lyase (tnaA) | 1483489 | 1484868 | carbon-carbon lyase activity | | | 0.2 |  |
| antisense: PG1401 |  |  |  |  | | | 0.21 |  |
| antisense: PG0263 | tyrosyl-tRNA synthetase (tyrS) | 295803 | 297095 |  | | | 0.21 |  |
| **Differential gene expression between early lysis and mid-stationary in W83 strain**  **analyzed by Rockhopper** | | | | | | | |  |
| **predicted asRNA target genes** | **Gene name** | **Locus region of gene** | | **Transcriptional location of predicted asRNA** | | **Predicted function/pathway** | **Fold changes** |  |
|  |  | **Start** | **Stop** | **Start** | **Stop** |  |  |  |
| antisense: PG1551 | hmuY | 1628578 | 1629006 | 1629012 | 1628355 |  | 0.16 |  |
| antisense: PG0933 | translation elongation factor G, putative | 990137 | 992296 | 991654 | 991636 |  | 0.6 |  |
| antisense: PG1858 | flavodoxin | 1956628 | 1957125 | 1956678 | 1957112 | iron ion binding | 0.11 |  |
| **Differential gene expression between late exponential phase of W83**  **and the same point (12.5hr) of W50 analyzed by Rockhopper** | | | | | | | |  |
| **predicted asRNA target genes** | **Gene name** | **Locus region of gene** | | **Transcriptional location of predicted asRNA** | | **Predicted function/pathway** | **Fold changes** |  |
|  |  | **Start** | **Stop** | **Start** | **Stop** |  |  |  |
| antisense: PG0537 | aminoacyl-histidine dipeptidase (pepD-2) | 586994 | 588454 | 588281 | 587153 |  | 4.2 |  |
| antisense: PG0195 | rubrerythrin | 232186 | 232764 | 232645 | 232206 | transition metal ion binding | 8 |  |
| antisense: PG1421 | ferredoxin | 1506964 | 1507134 | 1506940 | 1507176 | iron-sulphur binding | 10.5 |  |
| antisense: PG1616 | succinate dehydrogenase (or fumarate reductase) cytochrome b subunit, b558 family | 1696648 | 1697340 | 1697044 | 1697255 | succinate dehydrogenase activity (EC) | 2 |  |
| antisense: PG1941 | ribosomal protein S7 (rpsG) | 2027323 | 2027799 | 2027337 | 2027350 | structural constituent of ribosome | 0.35 |  |
| antisense: PG1615 | Succinate dehydrogenase/fumarate reductase, flavoprotein subunit (sdhA), fumarate reductase, flavoprotein subunit (frdA) | 1694674 | 1696617 | 1695347 | 1695364 | Fumarate reductase/succinate dehydrogenase flavoprotein-like | 2 |  |
| antisense: PG1615 |  |  |  | 1696288 | 1696480 |  | 2 |  |
| antisense: PG1615 |  |  |  | 1695760 | 1696063 |  | 2 (*q*<0.05) |  |
