# Supplementary material for: Growth of Porphyromonas gingivalis on human serum albumin triggers programmed cell death
Source: J Oral Microbiol. 2022 Dec 22;15(1):2161182. doi: 10.1080/20002297.2022.2161182 (PMC9788703; doi:10.1080/20002297.2022.2161182)
Supplement: Supplemental Material [file ZJOM_A_2161182_SM7719.zip › supplementary files/Supplemental Table S3a _Pathway Class (1).docx]

**Supplemental Table S3a (Pathway Class).** Differential gene expression analyzed by pairwise comparison of the transcriptomes of strains **W83 and W50 at 12.5hrs** (exponential phase of growth). Gene number, predicted function, and category (Pathway Class) are provided.

(Fold change ≥ 2; *q*-value < 0.01)

| **ID** | **Annotation** | **PathwayClass** | **logFC** |
| --- | --- | --- | --- |
| PG1837 | DUF2436 domain-containing protein | Cellular Processes; Cellular community | 1.58 |
| PG0514 | preprotein translocase subunit SecA | Membrane transport | -1.85 |
| PG1115 | signal recognition particle protein | Membrane transport | 1.41 |
| PG0598 | YjgP/YjgQ family permease | Membrane transport | 4.25 |
| PG2190 | ATP-binding cassette domain-containing protein | Membrane transport | 1.24 |
| PG1780 | Serine palmitoyl-transferase | Lipid metabolism | 2.32 |
| PG0468 | mannose-6-phosphate isomerase | Glycan biosynthesis and metabolism | 1.40 |
| PG1143 | UDP-glucose/GDP-mannose dehydrogenase | Glycan biosynthesis and metabolism | 1.78 |
| PG1277 | nucleotide sugar dehydrogenase | Glycan biosynthesis and metabolism | 4.67 |
| PG1288 | GDP-mannose 4 2C6-dehydratase | Glycan biosynthesis and metabolism | 1.96 |
| PG1342 | UDP-N-acetylmuramate dehydrogenase | Glycan biosynthesis and metabolism | 1.17 |
| PG0043 | family 20 glycosylhydrolase | Glycan biosynthesis and metabolism | 4.58 |
| PG0577 | phospho-N-acetylmuramoyl-pentapeptide-transferase | Glycan biosynthesis and metabolism | 1.65 |
| PG0581 | UDP-N-acetylmuramate--L-alanine ligase | Glycan biosynthesis and metabolism | 1.09 |
| PG1743 | 3-deoxy-8-phosphooctulonate synthase | Glycan biosynthesis and metabolism | 3.82 |
| PG1884 | alpha-L-fucosidase | Glycan biosynthesis and metabolism | -1.03 |
| PG0578 | UDP-N-acetylmuramoyl-L-alanine--D-glutamate ligase | Glycan biosynthesis and metabolism | 4.12 |
| PG0577 | phospho-N-acetylmuramoyl-pentapeptide-transferase | Glycan biosynthesis and metabolism | 1.65 |
| PG0581 | UDP-N-acetylmuramate--L-alanine ligase | Glycan biosynthesis and metabolism | 1.09 |
| PG1743 | 3-deoxy-8-phosphooctulonate synthase | Glycan biosynthesis and metabolism | 3.82 |
| PG1884 | alpha-L-fucosidase | Glycan biosynthesis and metabolism | -1.03 |
| PG0578 | UDP-N-acetylmuramoyl-L-alanine--D-glutamate ligase | Glycan biosynthesis and metabolism | 4.12 |
| PG0001 | chromosomal replication initiator protein DnaA | Genetic Information Processing; Folding, sorting and degradation | 1.53 |
| PG0401 | ribonuclease Y | Genetic Information Processing; Folding, sorting and degradation | 1.21 |
| PG0766 | polyribonucleotide nucleotidyltransferase | Genetic Information Processing; Folding, sorting and degradation | 1.32 |
| PG1208 | molecular chaperone DnaK | Genetic Information Processing; Folding, sorting and degradation | 1.25 |
| PG1721 | ribonuclease R | Genetic Information Processing; Folding, sorting and degradation | 3.09 |
| PG2099 | DEAD/DEAH box helicase | Genetic Information Processing; Folding, sorting and degradation | 2.54 |
| PG0271 | single-stranded DNA-binding protein | Genetic Information Processing; Replication and repair | 2.93 |
| PG1794 | DNA polymerase I | Genetic Information Processing; Replication and repair | 4.37 |
| PG1853 | DNA polymerase III subunit beta | Genetic Information Processing; Replication and repair | 1.05 |
| PG0394 | DNA-directed RNA polymerase subunit beta | Genetic Information Processing; Transcription | -1.25 |
| PG0395 | DNA-directed RNA polymerase subunit beta' | Genetic Information Processing; Transcription | -1.19 |
| PG0099 | phenylalanine--tRNA ligase subunit beta | Translation | 1.29 |
| PG0393 | 50S ribosomal protein L7/L12 | Translation | -1.17 |
| PG0962 | proline--tRNA ligase | Translation | 1.30 |
| PG0992 | threonine--tRNA ligase | Translation | 1.76 |
| PG1596 | isoleucine--tRNA ligase | Translation | 2.26 |
| PG1878 | cysteine--tRNA ligase | Translation | 2.63 |
| PG1951 | glutamine--tRNA ligase/YqeY | Translation | 2.02 |
| PG1960 | 50S ribosomal protein L28 | Translation | 2.11 |
| PG0143 | carbon-nitrogen hydrolase | Metabolism; Amino acid metabolism | 2.53 |
| PG0144 | agmatine deiminase family protein | Metabolism; Amino acid metabolism | 3.64 |
| PG1068 | 3-keto-5-aminohexanoate cleavage protein | Metabolism; Amino acid metabolism | 1.08 |
| PG1136 | asparagine synthetase B family protein | Metabolism; Amino acid metabolism | 3.82 |
| PG1741 | aspartate ammonia-lyase | Metabolism; Amino acid metabolism | 1.28 |
| PG2189 | aspartate kinase | Metabolism; Amino acid metabolism | 1.18 |
| PG1613 | methylmalonyl-CoA epimerase | Metabolism; Amino acid metabolism | 1.17 |
| PG1656 | methylmalonyl-CoA mutase small subunit | Metabolism; Amino acid metabolism | 2.85 |
| PG1657 | methylmalonyl-CoA mutase | Metabolism; Amino acid metabolism | 3.75 |
| PG1949 | malate dehydrogenase | Metabolism; Amino acid metabolism | 1.06 |
| PG0042 | serine hydroxymethyltransferase | Metabolism; Amino acid metabolism | 1.50 |
| PG0343 | methionine gamma-lyase | Metabolism; Amino acid metabolism | 2.52 |
| PG2121 | asparaginase | Metabolism; Amino acid metabolism | 4.21 |
| PG2188 | diaminopimelate decarboxylase | Metabolism; Amino acid metabolism | 1.74 |
| PG1114 | aspartate 1-decarboxylase | Metabolism; Metabolism of other amino acids | 3.82 |
| PG0137 | aminoacyl-histidine dipeptidase | Metabolism; Metabolism of other amino acids | 1.32 |
| PG0537 | aminoacyl-histidine dipeptidase | Metabolism; Metabolism of other amino acids | 5.91 |
| PG0249 | oxaloacetate decarboxylase | Metabolism; Carbohydrate metabolism | 1.73 |
| PG0429 | 2-oxoacid:acceptor oxidoreductase subunit alpha | Metabolism; Carbohydrate metabolism | 2.82 |
| PG0430 | 2-oxoacid:ferredoxin oxidoreductase subunit beta | Metabolism; Carbohydrate metabolism | 1.79 |
| PG0548 | pyruvate:ferredoxin (flavodoxin) oxidoreductase | Metabolism; Carbohydrate metabolism | 1.13 |
| PG1633 | galactokinase | Metabolism; Carbohydrate metabolism | 1.29 |
| PG1676 | phosphoenolpyruvate carboxykinase (ATP) | Metabolism; Carbohydrate metabolism | 1.57 |
| PG1677 | phosphoglycerate kinase | Metabolism; Carbohydrate metabolism | 3.30 |
| PG1682 | glycosyltransferase | Metabolism; Carbohydrate metabolism | 2.08 |
| PG1683 | alpha-amylase | Metabolism; Carbohydrate metabolism | 1.59 |
| PG1748 | transketolase | Metabolism; Carbohydrate metabolism | 1.80 |
| PG1809 | 2-oxoglutarate ferredoxin oxidoreductase | Metabolism; Carbohydrate metabolism | 2.49 |
| PG1810 | 2-oxoglutarate oxidoreductase | Metabolism; Carbohydrate metabolism | 2.86 |
| PG1812 | 3-methyl-2-oxobutanoate dehydrogenase | Metabolism; Carbohydrate metabolism | 2.45 |
| PG1813 | 4Fe-4S dicluster domain-containing protein | Metabolism; Carbohydrate metabolism | 2.79 |
| PG1996 | deoxyribose-phosphate aldolase | Metabolism; Carbohydrate metabolism | -1.26 |
| PG1608 | sodium ion-translocating decarboxylase | Metabolism; Carbohydrate metabolism | 1.17 |
| PG0793 | fructose-bisphosphatase class III | Metabolism; Energy metabolism | 1.01 |
| PG1614 | succinate dehydrogenase/fumarate reductase | Metabolism; Energy metabolism | 1.76 |
| PG1615 | fumarate reductase/succinate dehydrogenase | Metabolism; Energy metabolism | 2.66 |
| PG1616 | succinate dehydrogenase/fumarate reductase | Metabolism; Energy metabolism | 2.89 |
| PG1804 | V-type ATP synthase subunit B | Metabolism; Energy metabolism | -2.61 |
| PG1807 | ATP synthase subunit C | Metabolism; Energy metabolism | -1.44 |
| PG1820 | ammonia-forming cytochrome c nitrite reductase | Metabolism; Energy metabolism | 2.03 |
| PG1821 | cytochrome c nitrite reductase small subunit | Metabolism; Energy metabolism | 2.22 |
| PG1321 | formate--tetrahydrofolate ligase | Metabolism of cofactors and vitamins | -1.08 |
| PG1553 | cobaltochelatase subunit CobN | Metabolism of cofactors and vitamins | -1.13 |
| PG2109 | thiamine phosphate synthase | Metabolism of cofactors and vitamins | 4.22 |
| PG2205 | 2-dehydropantoate 2-reductase | Metabolism of cofactors and vitamins | -1.12 |
| PG2217 | 1-deoxy-D-xylulose-5-phosphate synthase | Metabolism of cofactors and vitamins | 2.14 |
| PG0784 | polyprenyl synthetase family protein | Metabolism of terpenoids and polyketides | 2.41 |
| PG0558 | purine nucleoside phosphorylase I | Metabolism; Nucleotide metabolism | -1.17 |
| PG0171 | bifunctional metallophosphosphatase/5'-nucleotidase | Metabolism; Nucleotide metabolism | 4.22 |
| PG0925 | thymidine kinase | Metabolism; Nucleotide metabolism | -1.47 |
| PG1353 | orotate phosphoribosyltransferase | Metabolism; Nucleotide metabolism | 1.47 |
| PG1530 | GTP pyrophosphokinase | Metabolism; Nucleotide metabolism | 2.53 |
| PG1648 | bifunctional (p)ppGpp synthetase/hydrolase | Metabolism; Nucleotide metabolism | 2.92 |
| PG1808 | bifunctional (p)ppGpp synthetase/hydrolase | Metabolism; Nucleotide metabolism | 4.78 |
