# Supplementary material for: Growth of Porphyromonas gingivalis on human serum albumin triggers programmed cell death
Source: J Oral Microbiol. 2022 Dec 22;15(1):2161182. doi: 10.1080/20002297.2022.2161182 (PMC9788703; doi:10.1080/20002297.2022.2161182)
Supplement: Supplemental Material [file ZJOM_A_2161182_SM7719.zip › supplementary files/Supplemental Table S3b _Pathway Class.docx]

**Supplemental Table S3b (Pathway Class).** Differential gene expression analyzed by pairwise comparison of the transcriptomes of strain **W83 (12.5hr)** with **W50 (20hr**); both in late exponential phase of growth. Gene number, predicted function, and pathway class are provided. (Fold change ≥ 2; *q*-value < 0.01)

| **ID** | **Annotation** | **Pathway Class** | **logFC** |
| --- | --- | --- | --- |
| PG0594 | RNA polymerase sigma factor RpoD/SigA | Genetic Information Processing; Transcription | -1.04 |
| PG1837 | DUF2436 domain-containing protein | Cellular Processes; Cellular community | 1.19 |
| PG0514 | preprotein translocase subunit SecA | Membrane transport | -2.55 |
| PG1667 | TolC family protein | Membrane transport | -2.62 |
| PG1654 | M15 family metallopeptidase | Membrane transport | -1.26 |
| PG1598 | lipoprotein signal peptidase | Membrane transport | 3.33 |
| PG0046 | phosphatidate cytidylyltransferase | Lipid metabolism | -1.55 |
| PG2141 | ketoacyl-ACP synthase III | Lipid metabolism | 1.23 |
| PG1239 | 3-oxoacyl-[acyl-carrier-protein] reductase | Lipid metabolism | 1.23 |
| PG1780 | Serine palmitoyl transferase | Lipid metabolism | 5.50 |
| PG1082 | acyltransferase | Lipid metabolism | 1.09 |
| PG0332 | transcription termination factor Rho | Genetic Information Processing; Folding, sorting and degradation | -2.19 |
| PG0520 | chaperonin GroEL | Genetic Information Processing; Folding, sorting and degradation | -1.85 |
| PG1208 | molecular chaperone DnaK | Genetic Information Processing; Folding, sorting and degradation | -1.62 |
| PG1721 | ribonuclease R | Genetic Information Processing; Replication and repair | 1.57 |
| PG0269 | exodeoxyribonuclease III | Genetic Information Processing; Replication and repair | -1.64 |
| PG0271 | single-stranded DNA-binding protein | Genetic Information Processing; Replication and repair | 2.27 |
| PG0811 | Holliday junction branch migration protein RuvA | Genetic Information Processing; Replication and repair | -1.47 |
| PG1072 | DNA mismatch repair protein MutS | Genetic Information Processing; Replication and repair | 3.58 |
| PG0001 | chromosomal replication initiator protein DnaA | Genetic Information Processing; Replication and repair | 1.63 |
| PG1911 | DNA-directed RNA polymerase | Translation | 1.31 |
| PG0099 | phenylalanine--tRNA ligase subunit beta | Translation | 1.25 |
| PG0263 | tyrosine--tRNA ligase | Translation | -1.68 |
| PG0315 | 50S ribosomal protein L27 | Translation | -1.05 |
| PG0592 | type B 50S ribosomal protein L31 | Translation | 4.02 |
| PG0992 | threonine--tRNA ligase | Translation | 3.12 |
| PG1297 | 30S ribosomal protein S1 | Translation | 1.00 |
| PG1596 | isoleucine--tRNA ligase | Translation | 2.23 |
| PG1723 | 30S ribosomal protein S20 | Translation | 2.85 |
| PG1878 | cysteine--tRNA ligase | Translation | 1.61 |
| PG1910 | 50S ribosomal protein L17 | Translation | 1.50 |
| PG1912 | 30S ribosomal protein S4 | Translation | 1.13 |
| PG1913 | 30S ribosomal protein S11 | Translation | 1.19 |
| PG1914 | 30S ribosomal protein S13 | Translation | 2.22 |
| PG1915 | 50S ribosomal protein L36 | Translation | 1.55 |
| PG1920 | 50S ribosomal protein L30 | Translation | 1.93 |
| PG1925 | 30S ribosomal protein S14 | Translation | 1.20 |
| PG1938 | 50S ribosomal protein L3 | Translation | 1.07 |
| PG1941 | 30S ribosomal protein S7 | Translation | 2.63 |
| PG1960 | 50S ribosomal protein L28 | Translation | 2.54 |
| PG2085 | tryptophan--tRNA ligase | Translation | 1.62 |
| PG0537 | aminoacyl-histidine dipeptidase | Metabolism of other amino acids | 5.59 |
| PG0084 | L-serine ammonia-lyase | Metabolism; Amino acid metabolism | -1.54 |
| PG0144 | agmatine deiminase family protein | Metabolism; Amino acid metabolism | 3.69 |
| PG0328 | imidazolonepropionase | Metabolism; Amino acid metabolism | -2.40 |
| PG1067 | hypothetical protein | Metabolism; Amino acid metabolism | 1.73 |
| PG1136 | asparagine synthetase B family protein | Metabolism; Amino acid metabolism | 3.87 |
| PG1269 | L-glutamate gamma-semialdehyde dehydrogenase | Metabolism; Amino acid metabolism | -1.55 |
| PG1424 | peptidylarginine deiminase PPAD | Metabolism; Amino acid metabolism | 1.99 |
| PG1741 | aspartate ammonia-lyase | Metabolism; Amino acid metabolism | 1.05 |
| PG1944 | 3-phosphoshikimate 1-carboxyvinyltransferase | Metabolism; Amino acid metabolism | -1.48 |
| PG2189 | aspartate kinase | Metabolism; Amino acid metabolism | 1.34 |
| PG1305 | aminomethyl-transferring glycine dehydrogenase | Metabolism; Amino acid metabolism | -1.39 |
| PG1613 | methylmalonyl-CoA epimerase | Metabolism; Amino acid metabolism | 2.00 |
| PG1656 | methylmalonyl-CoA mutase small subunit | Metabolism; Amino acid metabolism | 4.72 |
| PG2033 | NADPH-dependent glutamate synthase | Metabolism; Amino acid metabolism | 3.12 |
| PG0529 | glutamine-hydrolyzing carbamoyl-phosphate synthase | Metabolism; Amino acid metabolism | -1.21 |
| PG0249 | oxaloacetate decarboxylase | Metabolism; Carbohydrate metabolism | 1.18 |
| PG0429 | 2-oxoacid:acceptor oxidoreductase subunit alpha | Metabolism; Carbohydrate metabolism | 6.90 |
| PG0430 | 2-oxoacid:ferredoxin oxidoreductase subunit beta | Metabolism; Carbohydrate metabolism | 3.67 |
| PG0548 | pyruvate:ferredoxin (flavodoxin) oxidoreductase | Metabolism; Carbohydrate metabolism | 1.13 |
| PG0690 | acetyl-CoA hydrolase/transferase family protein | Metabolism; Carbohydrate metabolism | 1.12 |
| PG1017 | pyruvate 2C phosphate dikinase | Metabolism; Carbohydrate metabolism | -1.05 |
| PG1676 | phosphoenolpyruvate carboxykinase (ATP) | Metabolism; Carbohydrate metabolism | 1.60 |
| PG1677 | phosphoglycerate kinase | Metabolism; Carbohydrate metabolism | 5.19 |
| PG1682 | glycosyltransferase | Metabolism; Carbohydrate metabolism | 1.27 |
| PG1683 | alpha-amylase | Metabolism; Carbohydrate metabolism | 1.58 |
| PG1809 | 2-oxoglutarate ferredoxin oxidoreductase | Metabolism; Carbohydrate metabolism | 1.54 |
| PG1810 | 2-oxoglutarate oxidoreductase | Metabolism; Carbohydrate metabolism | 1.94 |
| PG1812 | 3-methyl-2-oxobutanoate dehydrogenase VorB | Metabolism; Carbohydrate metabolism | 1.43 |
| PG1813 | 4Fe-4S dicluster domain-containing protein | Metabolism; Carbohydrate metabolism | 2.14 |
| PG1996 | deoxyribose-phosphate aldolase | Metabolism; Carbohydrate metabolism | -2.28 |
| PG1615 | fumarate reductase/succinate dehydrogenase | Metabolism; Energy metabolism | 1.11 |
| PG1616 | succinate dehydrogenase/fumarate reductase | Metabolism; Energy metabolism | 2.06 |
| PG1801 | V-type ATP synthase | Metabolism; Energy metabolism | 2.76 |
| PG1804 | V-type ATP synthase subunit B | Metabolism; Energy metabolism | -3.06 |
| PG1820 | ammonia-forming cytochrome c nitrite reductase | Metabolism; Energy metabolism | 1.71 |
| PG1821 | cytochrome c nitrite reductase small subunit | Metabolism; Energy metabolism | 1.56 |
| PG1998 | polyprenyl synthetase family protein | Metabolism of terpenoids and polyketides | 1.68 |
| PG1143 | UDP-glucose/GDP-mannose dehydrogenase | Metabolism; Glycan biosynthesis and metabolism | 2.11 |
| PG1277 | nucleotide sugar dehydrogenase | Metabolism; Glycan biosynthesis and metabolism | 1.68 |
| PG2215 | mannose-1-phosphate guanylyltransferase | Metabolism; Glycan biosynthesis and metabolism | -1.30 |
| PG0087 | SIS domain-containing protein | Metabolism; Glycan biosynthesis and metabolism | 3.67 |
| PG1815 | 3-deoxy-manno-octulosonate cytidylyltransferase | Metabolism; Glycan biosynthesis and metabolism | 3.69 |
| PG1884 | alpha-L-fucosidase | Metabolism; Glycan biosynthesis and metabolism | -1.87 |
| PG0057 | nicotinate phosphoribosyltransferase | Metabolism of cofactors and vitamins | 1.14 |
| PG0625 | GTP cyclohydrolase I FolE | Metabolism of cofactors and vitamins | -1.02 |
| PG1321 | formate--tetrahydrofolate ligase | Metabolism of cofactors and vitamins | -1.30 |
| PG2205 | 2-dehydropantoate 2-reductase | Metabolism of cofactors and vitamins | -1.51 |
| PG0558 | purine nucleoside phosphorylase | Metabolism; Nucleotide metabolism | -1.22 |
| PG2097 | ribose-phosphate pyrophosphokinase | Metabolism; Nucleotide metabolism | 1.90 |
| PG1353 | orotate phosphoribosyltransferase | Metabolism; Nucleotide metabolism | 1.25 |
| PG1530 | GTP pyrophosphokinase | Metabolism; Nucleotide metabolism | 2.65 |
| PG1648 | bifunctional (p)ppGpp synthetase/ hydrolase | Metabolism; Nucleotide metabolism | 2.24 |
| PG1808 | bifunctional (p)ppGpp synthetase/ hydrolase | Metabolism; Nucleotide metabolism | 4.84 |
